# *In vitro* functional characterization of *Tadarida brasiliensis* interferon-β: promoter responsiveness, antiviral activity, and JAK-dependent transcriptional responses

**DOI:** 10.3389/fimmu.2026.1915149

**Published:** 2026-09-03

**Authors:** Xingyu Liu, Shuhan Li, Weijia Chen, Jie Wang, Qiuju Liu, Feiyu Fu, Qi Shao, Zhaofei Wang, Jingjiao Ma, Hengan Wang, Yaxian Yan, Yuqiang Cheng, Jianhe Sun

**Affiliations:** 1 Shanghai Key Laboratory of Veterinary Biotechnology, School of Agriculture and Biology, Shanghai Jiao Tong University, Shanghai, China; 2 Shanghai Jiao Tong University School of Medicine, Shanghai, China; 3 Shanghai Institute of Infectious Disease and Biosecurity, Fudan University, Shanghai, China

**Keywords:** antiviral activity, innate immunity, interferon-β, promoter, *Tadarida brasiliensis*

## Abstract

**Introduction:**

Bats display diverse antiviral responses, but interferon-β remains poorly characterized in many bat species. Here, we characterized interferon-β from *Tadarida brasiliensis* (TBIFN-β) in Tb 1 Lu cells, focusing on its sequence features, promoter responsiveness, antiviral activity, and downstream signaling.

**Methods:**

TBIFN-β was analyzed by comparative sequence analysis and homology modeling. TBIFNB promoter fragments were evaluated using dual-luciferase assays following batIRF1 expression or poly(I:C) treatment. Antiviral activity was assessed using VSV-GFP and VACV-GFP infection models, together with viral transcript quantification by RT-qPCR. Downstream signaling was examined through interferon-stimulated gene expression, Pyridone 6-mediated JAK inhibition, conditioned-medium stimulation, STAT1 phosphorylation, and exploratory RNA sequencing followed by GO/KEGG enrichment analysis and RT-qPCR validation.

**Results:**

TBIFN-β retained the principal predicted structural features of mammalian type I interferons. VSV-GFP and VACV-GFP infection induced distinct temporal changes in endogenous TBIFNB and interferon-stimulated gene expression. Both −900/0 and −500/0 TBIFNB promoter fragments responded to batIRF1 and poly(I:C). Transfection with the TBIFN-β expression construct reduced VSV-GFP and VACV-GFP fluorescence and decreased VSV-NP and VACV-C23L transcript copy equivalents. TBIFN-β induced context-dependent expression of PKR, OAS1, and Mx-1, with OAS1 showing the most consistent response. Pyridone 6 attenuated TBIFN-β-associated STAT1 and ISG responses and partially reduced its antiviral effects. Conditioned medium containing Flag-tagged TBIFN-β induced Pyridone 6-sensitive STAT1 phosphorylation in recipient cells. Transcriptomic analysis further revealed coordinated interferon-associated and antiviral transcriptional responses following TBIFN-β overexpression.

**Discussion:**

These findings provide an integrated *in vitro* characterization of TBIFN-β promoter responsiveness, antiviral activity, and JAK-dependent transcriptional effects in Tb 1 Lu cells. The results establish a basis for further functional studies of *T. brasiliensis* interferon biology, while validation in primary bat cells and in vivo models remains necessary.

## 1 Introduction

Bats are natural hosts of diverse viruses, including members of several viral families associated with disease in humans and domestic animals (1, 2). In some natural and experimental infection settings, bat species can support viral replication without developing the severe clinical manifestations observed in other mammals. This phenotype has been associated with a combination of ecological, physiological, and immunological characteristics rather than a single universal mechanism (1, 2). Recent comparative genomic studies have identified extensive adaptation of genes involved in viral recognition, interferon signaling, inflammation, and antiviral effector functions in bats (3, 4). However, these adaptations are not uniform across Chiroptera. Considerable variation exists among bat lineages, emphasizing the need to investigate antiviral immunity at the species level rather than assuming a common pan-bat response.

Type I interferons are central components of the mammalian antiviral response. Recognition of viral nucleic acids by cellular pattern-recognition receptors activates interferon regulatory factors and other transcription factors, leading to the production of type I interferons. Among these cytokines, interferon beta (IFN-β) is an important early mediator of antiviral defense. Following secretion, IFN-β binds to the type I interferon receptor complex and activates downstream signaling pathways, particularly the Janus kinase and signal transducer and activator of transcription pathway. This response promotes the expression of interferon-stimulated genes that restrict viral entry, genome replication, protein synthesis, assembly, or release. Although the principal architecture of type I interferon signaling is conserved among mammals, IFN-β can exhibit receptor-binding and signaling properties that differ from those of other type I interferon subtypes (5).

Accumulating evidence indicates that bat type I interferon systems contain both conserved and species-associated features. Comparative analyses have identified differences in the organization and composition of type I interferon loci, including lineage-dependent changes in IFN-α and IFN-ω-like genes (4, 6). Functional studies using cells from *Pteropus alecto* and *Eptesicus fuscus* have shown that species-matched IFN-β activates canonical interferon-stimulated genes and restricts viral infection, while also revealing differences in signaling and effector responses between species. Bat interferon regulatory factors also retain important antiviral functions. IRF1 and IRF7 directly regulate interferon-associated genes in *P. alecto* (7), while IRF3 contributes to poly(I:C)- and virus-induced IFN-β expression in insectivorous bat cells (5, 7, 8). More recently, IRF7 from *Tadarida brasiliensis* was shown to respond to viral infection and poly(I:C) stimulation and to activate antiviral innate immune responses in Tb 1 Lu cells (8). Together, these studies demonstrate that functional antiviral pathways are present in bats but may differ in their regulation, magnitude, and downstream gene profiles among species.

Despite these advances, IFN-β has been functionally characterized in only a limited number of bat species. The sequence features, predicted structure, promoter regulation, downstream signaling, and antiviral activity of IFN-β from *T. brasiliensis* remain incompletely defined. This knowledge gap is particularly relevant because results obtained in fruit bats or other insectivorous bat lineages cannot necessarily be extrapolated to *T. brasiliensis*. In addition, although IRF7 has been characterized in Tb 1 Lu cells, the regulatory architecture of the corresponding TBIFNB promoter and its responsiveness to bat transcription factors and viral nucleic acid analogues have not been systematically evaluated.

In the present study, we cloned and characterized the IFN-β coding sequence from *T. brasiliensis* and examined its phylogenetic relationships, amino acid variation, and predicted three-dimensional structure. Particular attention was given to residue 48 because this position varied among the mammalian sequences analyzed. However, its functional significance was not assumed from sequence or structural modeling alone. We also characterized the upstream regulatory region of TBIFNB, generated truncated promoter reporter constructs, and evaluated their responses to batIRF1 expression and poly(I:C) treatment. Parallel human IFNB promoter reporter experiments were included as descriptive benchmarks rather than as direct measurements of intrinsic promoter sensitivity.

We further examined the effects associated with transfection of a TBIFN-β expression construct in Tb 1 Lu cells using several RNA and DNA virus infection models. Downstream responses were assessed by measuring selected interferon-stimulated genes, analyzing ISG promoter activity, pharmacologically inhibiting JAK signaling with Pyridone 6, and performing exploratory transcriptomic analysis. This integrated approach was designed to define the cellular activity and regulatory characteristics of TBIFN-β under the tested *in vitro* conditions. Because the study was conducted using one immortalized cell line from a single bat species, the findings were not intended to establish a general mechanism of viral tolerance or asymptomatic viral carriage across bats.

## 2 Materials and methods

### 2.1 Cell lines and culture conditions

Tb 1 Lu cells (ATCC CCL-88), an epithelial-like cell line derived from the lung of an adult female *Tadarida brasiliensis*, were obtained from the American Type Culture Collection. HEK293T cells and HeLa cells were obtained from the American Type Culture Collection. Cells were maintained in Dulbecco’s modified Eagle medium supplemented with 10% fetal bovine serum at 37 °C in a humidified incubator containing 5% CO_2_. Cells were passaged using 0.25% trypsin-EDTA when they reached approximately 80% to 90% confluence.

### 2.2 Viruses

Influenza A virus subtype H1N1 (IAV-H1N1) and green fluorescent protein-expressing vaccinia virus (VACV-GFP) were maintained in our laboratory. Green fluorescent protein-expressing pseudorabies virus (PRV-GFP) was a gift from Professor Beibei Chu of Henan Agricultural University, and green fluorescent protein-expressing vesicular stomatitis virus (VSV-GFP) was kindly provided by Professor Tao Sun of Shanghai Jiao Tong University. All viruses were propagated in Tb 1 Lu cells. Viral titers were determined using the 50% tissue culture infectious dose (TCID_50_) method. Viral stocks were stored at −80 °C at the Shanghai Key Laboratory of Veterinary Biotechnology, School of Agriculture and Biology, Shanghai Jiao Tong University, Shanghai, China.

### 2.3 Reagents, antibodies, and kits

Dulbecco’s modified Eagle medium (DMEM; catalog no. C11995500CP), fetal bovine serum (FBS; catalog no. A5256701), and 0.25% trypsin-EDTA (catalog no. 25200-072) were purchased from Gibco, Thermo Fisher Scientific. Phosphate-buffered saline (PBS; pH 7.4) was purchased from Servicebio. PlusTrans™ Transfection Reagent was purchased from NULEN Biotech Co., Ltd. (catalog no. CT801). Low-molecular-weight polyinosinic-polycytidylic acid complexed with LyoVec™, Poly(I:C) (LMW)/LyoVec™, was purchased from InvivoGen (catalog no. tlrl-picwlv). Pyridone 6, also known as JAK inhibitor I or CMP 6, was purchased from Beyotime Biotechnology (catalog no. SD4758-10mM). Dimethyl sulfoxide (DMSO) was purchased from MP Biomedicals (catalog no. 196055). Total RNA was extracted using the VAMNE Magnetic Cell/Tissue Total RNA Kit (32 Prepackaged) (Vazyme Biotech Co., Ltd.; catalog no. RMA101-C2-P3). Complementary DNA was synthesized using HiScript III RT SuperMix with gDNA wiper (Vazyme; catalog no. R323-01). Quantitative PCR was performed using ChamQ Blue Universal SYBR qPCR Master Mix (Vazyme; catalog no. Q312-02). Conventional PCR amplification was performed using 2× Phanta Flash Master Mix (Dye Plus) (Vazyme; catalog no. P520-03-AA). Genomic DNA was isolated using the FastPure Cell/Tissue DNA Isolation Mini Kit (Vazyme; catalog no. DC102-01). PCR products were purified using the FastPure Gel DNA Extraction Mini Kit (Vazyme; catalog no. DC301-01) and cloned using the Ultra-Universal TOPO Cloning Kit (Vazyme; catalog no. C603-02). Endotoxin-free plasmid DNA was prepared using the Endo-Free Plasmid Mini Kit II (Omega Bio-Tek; catalog no. D6950-01). Firefly and Renilla luciferase activities were measured using the Firefly & Renilla-Light Luciferase Reporter Assay Kit (MeilunBio; catalog no. MA0518). Mild RIPA lysis buffer (P0013D) and QuickBlock™ Universal Protein-Free Blocking and Antibody Dilution Buffer (P0270) were purchased from Beyotime (Shanghai, China). The 5× SDS-PAGE Protein Loading Buffer (20315ES05), Super ECL Detection Reagent (36208ES76), HRP-conjugated goat anti-mouse IgG (H+L) (33201ES60), and HRP-conjugated goat anti-rabbit IgG (H+L) (33101ES60) were obtained from Yeasen Biotechnology (Shanghai, China). InStab™ Phosphatase Inhibitor Cocktail was also obtained from Yeasen Biotechnology. The primary antibodies used were rabbit anti-GAPDH antibody (Proteintech, 10494-1-AP), rabbit anti-phospho-STAT1 (Tyr701) antibody (Cell Signaling Technology, 7649T), rabbit anti-STAT1 antibody (Cell Signaling Technology, 9172T), and mouse anti-DYKDDDDK-tag (3B9) monoclonal antibody (Abmart, M20008). DAPI staining solution was purchased from Beyotime Biotechnology (catalog no. C1002). Unless otherwise specified, all reagents and kits were used according to the manufacturers’ instructions.

### 2.4 Virus infection

Tb 1 Lu cells were seeded into 12-well culture plates at a density of 2.5 × 10^4^ cells/well and when cultures reached 80% confluence, the culture medium was replaced with fresh serum-free DMEM, and infected with VSV-GFP or IAV-H1N1 at an MOI of 0.001 and VACV-GFP or PRV-GFP at an MOI of 0.1. After 6 h, 12 h, 24 h and 36h of infection, samples were collected for subsequent experiments.

### 2.5 Plasmid transfection

Tb 1 Lu cells were seeded in 24-well plates at a density of 5 × 10^5^ cells/mL in 500 μL of culture medium per well, corresponding to 2.5 × 10^5^ cells per well. For experiments performed in 12-well plates, cells were seeded at a density of 1 × 10^6^ cells/mL in 1 mL of culture medium per well, corresponding to 1 × 10^6^ cells per well. Cells were cultured at 37 °C in a humidified incubator containing 5% CO_2_. When the cells reached approximately 80% confluence, plasmid transfection was performed using PlusTrans™ Transfection Reagent. A total of 500 ng or 1000 ng of plasmid DNA was used per well of a 24-well or 12-well plate, respectively. Plasmid DNA and PlusTrans™ Transfection Reagent were mixed at a ratio of 1 μg DNA to 1.5 μL transfection reagent. The DNA-reagent complexes were incubated at room temperature for 20 min and then added to the cells. Cells were incubated for 24 h after transfection before viral infection or sample collection.

### 2.6 RNA extraction and cDNA synthesis

Total RNA was extracted from Tb 1 Lu cells collected after the indicated plasmid transfection, pharmacological treatment, or viral infection using the VAMNE Magnetic Cell/Tissue Total RNA Kit (32 Prepackaged) according to the manufacturer’s instructions. The concentration and purity of the extracted RNA were assessed using a spectrophotometer. Genomic DNA was removed using the gDNA wiper component of the HiScript III RT SuperMix with gDNA wiper kit. For each reverse-transcription reaction, 1 μg of total RNA was used to synthesize cDNA in a final reaction volume of 10 μL. The resulting cDNA was diluted to a final volume of 200 μL with nuclease-free water before subsequent qPCR analysis.

### 2.7 Quantitative real-time PCR

Quantitative real-time PCR was performed using ChamQ Blue Universal SYBR qPCR Master Mix on an ABI 7500 Real-Time PCR System (Applied Biosystems, Thermo Fisher Scientific). For each sample, 1,000 ng of total RNA was reverse transcribed in a final volume of 10 μL. The resulting cDNA was diluted to a final volume of 200 μL before qPCR analysis. Each qPCR reaction was performed in a final volume of 10 μL containing 5 μL of 2× ChamQ Blue Universal SYBR qPCR Master Mix, 0.3 μL of each forward and reverse primer at a stock concentration of 10 μM, and 4.4 μL of 20-fold-diluted cDNA. The final concentration of each primer was 0.3 μM. The amplification program consisted of an initial denaturation step at 95 °C for 5 min, followed by 40 cycles of denaturation at 95 °C for 10 s and annealing and extension at 60 °C for 30 s. Each biological sample was analyzed in three technical replicates. Primer sequences are provided in **Supplementary Table 1**. The amplification efficiencies of the host-gene primer pairs ranged from 90.1% to 108.9%, with coefficients of determination (R²) ranging from 0.9925 to 0.9997 (**Supplementary Table 5**). For host gene expression analysis, β-actin was used as the reference gene. Its stability under VSV-GFP and VACV-GFP infection was evaluated using raw Ct values obtained from three independent biological experiments across the corresponding infection time courses. β-actin showed limited Ct variation across the examined time points, with Ct ranges of 16.59–17.05 during VSV-GFP infection and 15.23–16.00 during VACV-GFP infection (**Supplementary Table 4**). Relative transcript abundance was calculated using the 2^−ΔΔCt method and expressed relative to the corresponding control group, including the 0 h, mock-treated, empty-vector, or vehicle-control group, as appropriate. Viral VSV-NP and VACV-C23L transcripts were quantified using external standard curves as described below and were not normalized to β-actin. Unless otherwise indicated, experiments were independently repeated three times.

### 2.8 Absolute quantification of viral transcript copy equivalents

Purified PCR products corresponding to a 183-bp fragment of VSV-NP and a 146-bp fragment of VACV-C23L were used to generate external standards for absolute quantification. Following measurement of the purified PCR product concentrations, the copy number concentration was calculated according to the molecular weight of each amplicon. Serial dilutions spanning 10^2^ to 10^7^ copies/μL were prepared and analyzed using the same qPCR conditions as those used for the experimental samples. Standard curves were generated by plotting the Ct values against the logarithm of the standard copy concentrations. The standard curve equations were Ct = −3.3335 log10(C) + 34.8332 for VSV-NP and Ct = −3.2647 log10(C) + 35.5060 for VACV-C23L, where C represents the copy concentration in copies/μL. The corresponding coefficients of determination were 0.9986 and 0.9885, and the calculated amplification efficiencies were 99.5% and 102.4%, respectively. Ct values obtained from the experimental samples were converted to viral transcript copy equivalents using the corresponding standard curve. Because the reverse-transcription products were diluted 20-fold before qPCR, the calculated concentrations were corrected by the dilution factor and expressed as copy equivalents per microliter of the original cDNA preparation. Viral transcript copy equivalents were not normalized to β-actin. Each sample was analyzed in three technical replicates, and three independent biological experiments were performed.

### 2.9 Cloning and sequence verification of TBIFN-β

Total RNA was extracted from Tb 1 Lu cells, and cDNA was synthesized as described above. Gene-specific primers were designed based on the predicted *Molossus molossus* IFNB sequence deposited in the NCBI database (Gene ID: 118621468; RefSeq accession no. XM_036260365.1). The primer sequences are listed in **Supplementary Table 1**. The TBIFNB coding sequence was amplified from Tb 1 Lu cell cDNA using 2× Phanta Flash Master Mix (Dye Plus). The PCR program consisted of an initial denaturation step at 98 °C for 30 s, followed by 35 cycles of denaturation at 98 °C for 10 s, annealing at 58 °C for 5 s, and extension at 72 °C for 20 s, with a final extension at 72 °C for 1 min. PCR products were separated by electrophoresis on a 1% agarose gel. The DNA fragment of the expected size, approximately 561 bp, was excised and purified using the FastPure Gel DNA Extraction Mini Kit. The purified PCR product was cloned using the Ultra-Universal TOPO Cloning Kit. Positive clones were selected and subjected to Sanger sequencing by Tsingke Biotechnology Co., Ltd. (Beijing, China). The sequencing results were assembled and compared with the reference sequence using SnapGene version 6.0.2. The confirmed TBIFNB nucleotide sequence was translated into its predicted amino acid sequence and used for subsequent plasmid construction and comparative sequence analyses.

### 2.10 Comparative sequence, phylogenetic, and structural analyses

The confirmed TBIFNB coding sequence was translated into its deduced amino acid sequence using SnapGene version 6.0.2. IFN-β amino acid sequences from representative mammalian species, including selected bat species, were retrieved from the National Center for Biotechnology Information and UniProt databases. The species names, database sources, and accession numbers of all sequences used in the analyses are provided in **Supplementary Table 2**. Multiple amino acid sequence alignments were performed using the ClustalW algorithm implemented in MEGA version 5.1. Pairwise amino acid identity between TBIFN-β and representative IFN-β proteins was calculated from the full-length ClustalW alignment as the percentage of identical residues among aligned positions containing amino acids in both sequences, with gap-containing positions excluded. Separate phylogenetic trees were constructed for representative mammalian IFN-β proteins and bat IFN-β proteins using the maximum-likelihood method based on the Jones-Taylor-Thornton matrix-based model. The reliability of the tree topology was evaluated using 1,000 bootstrap replicates. Conserved domains in the predicted TBIFN-β protein were analyzed using the InterPro database. A three-dimensional model of TBIFN-β was generated using the SWISS-MODEL server, with the crystal structure of human IFN-β as the template (PDB ID: 1AU1, chain A). The predicted TBIFN-β structure and the human IFN-β structure were visualized and superimposed using PyMOL version 3.1.8. Structural similarity was evaluated using the root-mean-square deviation calculated from the aligned Cα atoms. These analyses were used to assess the overall similarity between the predicted protein structures and were not considered direct evidence of receptor binding or biological activity.

### 2.11 Construction of TBIFN-β expression plasmid

The 561-bp TBIFNB coding sequence without the stop codon was amplified and inserted in frame into the pcDNA3.1(+) expression vector containing a C-terminal Flag tag. The vector and TBIFNB fragment were digested with *EcoRI* and *XhoI* and subsequently ligated to generate the pcDNA3.1-TBIFN-β-Flag expression plasmid. The ligation products were transformed into *Escherichia coli* DH5α competent cells and selected on Luria-Bertani agar containing ampicillin. Five positive colonies were subjected to Sanger sequencing by Tsingke Biotechnology Co., Ltd. Sequence-confirmed plasmids were purified using the Endo-Free Plasmid Mini Kit II and stored at −20 °C until use.

### 2.12 TBIFN-β promoter analysis and dual-luciferase reporter assay

Genomic DNA was extracted from Tb 1 Lu cells, and a 2,000-bp region upstream of the TBIFNB translation initiation codon was amplified using primers designed based on the corresponding *Rhinolophus sinicus* (NW_017739229.1) reference genomic sequence. PCR was performed with an initial denaturation at 98 °C for 30 s, followed by 35 cycles of 98 °C for 10 s, 58 °C for 5 s, and 72 °C for 20 s, with a final extension at 72 °C for 1 min. The PCR product was separated on a 1% agarose gel, purified, and verified by Sanger sequencing. Putative transcription-factor-binding sites were predicted using PROMO, the Berkeley Drosophila Genome Project promoter prediction tool, and JASPAR. The −900/0 and −500/0 fragments were selected based on the predicted distribution of regulatory motifs within the upstream region. The −900/0 fragment encompassed both the more distal predicted NF-κB-associated region and proximal IRF-associated motifs, whereas the −500/0 fragment retained the proximal predicted IRF-binding sites while excluding the more distal region, allowing us to evaluate whether the proximal promoter region was sufficient to support transcriptional responsiveness. Two truncated promoter fragments spanning −900 to 0 bp and −500 to 0 bp relative to the translation initiation codon were amplified. The pGL3.0-basic vector was linearized by inverse PCR, and the promoter fragments were inserted upstream of the firefly luciferase gene by homologous recombination. The recombinant plasmids were transformed into *Escherichia coli* DH5α competent cells and selected on Luria-Bertani agar containing ampicillin. Five candidate positive colonies were subjected to Sanger sequencing. Sequence-confirmed plasmids were purified using the Endo-Free Plasmid Mini Kit II, quantified using a Nano-100 spectrophotometer, and stored at −20 °C. The resulting reporter plasmids were designated pGL3.0-batIFN-β-P(−900/0)-Luc and pGL3.0-batIFN-β-P(−500/0)-Luc.

### 2.13 Dual luciferase reporter assay

Tb 1 Lu cells were cultured in 24-well plates and transfected at approximately 80% confluence. For batIRF1-mediated promoter activation assays, cells were co-transfected with 120 ng of the indicated TBIFNB promoter reporter plasmid, 60 ng of pRL-TK, and 300 ng of pcDNA3.1-batIRF1-Flag per well. The pGL3.0-basic vector was used as the promoterless reporter control. For the initial poly(I:C)-mediated promoter activation assay, cells were transfected with 120 ng of the indicated TBIFNB promoter reporter plasmid and 60 ng of pRL-TK per well. Poly(I:C) (LMW)/LyoVec™ was added directly to the culture medium at 50 ng per well at the time of reporter transfection and was not included in the PlusTrans™ DNA-transfection complexes. Firefly and Renilla luciferase activities were measured 24 h later. For the poly(I:C) concentration-response assay, Tb 1 Lu cells transfected with the selected TBIFNB promoter reporter construct and pRL-TK were treated with poly(I:C) at final concentrations of 0, 0.5, or 1 μg/mL. Dual-luciferase activity was measured at 6 h after treatment. For the time-course assay, cells were treated with 1 μg/mL poly(I:C), and dual-luciferase activity was measured at 0, 6, 12, and 24 h after treatment. For descriptive benchmarking of human and bat IFNB promoter reporter responses, HEK293T and HeLa cells were transfected with the huIFNB promoter firefly luciferase reporter construct together with pRL-TK, whereas Tb 1 Lu cells were transfected with the corresponding TBIFNB promoter reporter construct together with pRL-TK. Cells were treated with 0, 0.5, or 1 μg/mL poly(I:C), and dual-luciferase activity was measured at 6, 12, and 24 h after treatment. Poly(I:C) was added directly to the culture medium at the start of the indicated treatment period. Firefly and Renilla luciferase activities were measured using the Firefly & Renilla-Light Luciferase Reporter Assay Kit with a Synergy LX multimode microplate reader (BioTek Instruments, Inc., USA). Relative promoter activity was calculated as the ratio of firefly to Renilla luciferase activity. For the concentration- and time-response experiments, the normalized activity of the corresponding untreated control was set to 1. For the ISG promoter reporter assays, Tb 1 Lu cells were co-transfected with 120 ng of the indicated PKR, Mx1, or OAS1 promoter firefly luciferase reporter construct, 60 ng of pRL-TK, and 300 ng of either the empty vector or pcDNA3.1-TBIFN-β-Flag per well. Firefly and Renilla luciferase activities were measured at 24 h after transfection. Relative promoter activity was calculated as the ratio of firefly to Renilla luciferase activity, and the activity of the corresponding empty-vector group was set to 1. Each condition was tested in three technical replicate wells, and all experiments were independently repeated three times.

### 2.14 Assessment of the antiviral activity of TBIFN-β

To evaluate the antiviral activity associated with TBIFN-β expression, Tb 1 Lu cells were transfected with either pcDNA3.1-TBIFN-β-Flag or the corresponding empty vector (EV). At 24 h after transfection, the cells were infected with VSV-GFP at a multiplicity of infection (MOI) of 0.001, VACV-GFP at an MOI of 0.1, PRV-GFP at an MOI of 0.1, or IAV-H1N1 at an MOI of 0.001. For VSV-GFP and VACV-GFP infections, GFP fluorescence images were acquired at the indicated time points using an EVOS M7000 inverted fluorescence microscope (Invitrogen). For DAPI-counterstained imaging, cells collected at 0, 24, and 36 h post-infection were fixed with 4% paraformaldehyde for 10 min and washed three times with PBS. The cells were then stained with 10 μg/mL DAPI for 15 min, followed by three washes with PBS. GFP fluorescence, DAPI-stained nuclei, and merged images were subsequently acquired. For GFP imaging, fixed acquisition settings were used within each virus experiment. The GFP-channel settings displayed by the EVOS M7000 software were an illumination-intensity setting of 0.070, an exposure setting of 0.0800, and a gain setting of 75.0. These settings were kept unchanged when images from the corresponding experimental groups were acquired. Fluorescence images presented in the main figure were acquired using a 10× objective, whereas the supplementary figures include images acquired using both 4× and 10× objectives. GFP fluorescence microscopy was used as a qualitative indicator of the distribution and progression of viral infection and was not subjected to numerical fluorescence-intensity quantification or normalization. Quantitative assessment of viral transcript abundance was based on VSV-NP and VACV-C23L transcript copy equivalents determined by RT-qPCR using external standard curves as described in Section 2.8. For PRV-GFP and IAV-H1N1 infections, cells were collected at 24 h post-infection, and the relative expression levels of PKR, OAS1, and Mx1 were measured by RT-qPCR. These transcript levels were normalized to β-actin and calculated using the 2^−ΔΔCt method, with the corresponding EV group used as the calibrator. All experiments were independently repeated three times.

### 2.15 Pyridone 6 inhibition assay

Tb 1 Lu cells cultured in 12-well plates were transfected with either the empty vector or pcDNA3.1-TBIFN-β-Flag. At 24 h after transfection, cells were treated with 10 μM Pyridone 6 or an equivalent volume of DMSO for 2 h. The final concentration of DMSO was 0.1% (v/v). Following pretreatment, cells were mock infected or infected with VSV-GFP or VACV-GFP under the conditions described above. The culture medium was not replaced, and Pyridone 6 or DMSO remained present throughout the infection period. For each infection condition, the experimental groups were empty vector plus DMSO, empty vector plus Pyridone 6, TBIFN-β plus DMSO, and TBIFN-β plus Pyridone 6. Cells were collected at 24 h post-infection, and total RNA was extracted for subsequent RT-qPCR analysis. Each experiment was independently repeated three times.

### 2.16 Preparation of conditioned medium and recipient-cell treatment

Tb 1 Lu cells were transfected with either the empty-vector plasmid or the TBIFNB-Flag expression plasmid. At 6 h after transfection, the medium was replaced with fresh culture medium. Conditioned medium was collected at 24 h after transfection and centrifuged at 1,000 rpm for 5 min to remove cells and debris. Recipient Tb 1 Lu cells were cultured in 12-well plates and treated at approximately 80% confluence. The original medium was removed, and the cells were washed twice with PBS. Cells were then pretreated with 1 mL of serum-free DMEM containing 10 μM Pyridone 6 for 2 h. Control cells received an equivalent volume of DMSO. The pretreatment medium was subsequently replaced with 1 mL of undiluted conditioned medium containing 10 μM Pyridone 6 or the corresponding volume of DMSO. Cells were collected at 0, 30, and 60 min after conditioned-medium addition. The 0-min samples were collected immediately after treatment.

### 2.17 Protein preparation and immunoblotting

For conditioned-medium protein preparation, 500 μL of clarified conditioned medium was mixed with 500 μL of methanol and 125 μL of chloroform, vortexed thoroughly, and centrifuged at 10,000 rpm for 5 min at 4 °C. After removal of the upper aqueous/methanol phase, 500 μL of methanol was added to the remaining material, followed by centrifugation at 10,000 rpm for 10 min at 4 °C. The supernatant was discarded, and the protein pellet was air-dried in a fume hood at room temperature for 10 min. The pellet was resuspended in 100 μL of 1× SDS-PAGE protein loading buffer and heated in a boiling water bath for 15 min.

For cellular protein extraction, Tb 1 Lu cells cultured in 12-well plates were lysed on ice for 15 min with 100 μL of mild RIPA lysis buffer supplemented with PMSF and phosphatase inhibitor cocktail, each at a 1:100 dilution. The lysates were centrifuged at 12,000 rpm for 10 min at 4 °C, and the clarified supernatants were collected. The lysates were mixed with 5× SDS-PAGE protein loading buffer at a lysate-to-buffer ratio of 4:1 and heated in a boiling water bath for 15 min. Protein concentrations were not measured. For both cellular lysates and conditioned-medium samples, 10 μL of each prepared sample was loaded per lane.

Proteins were separated by SDS-PAGE using 15% resolving gels for DYKDDDDK-tag detection and 10% resolving gels for STAT1, phospho-STAT1, and GAPDH detection. Electrophoresis was performed at 80 V for 30 min through the stacking gel, followed by 120 V for 2 h through the resolving gel. Proteins were transferred onto 0.2-μm PVDF membranes using a wet-transfer system at 100 V for 1 h. Before transfer, the membranes were activated in methanol for 30 s. After transfer, the membranes were cut horizontally according to the molecular-weight regions of the target proteins and blocked with QuickBlock™ buffer for 30 min at room temperature.

Primary antibodies were diluted in QuickBlock™ buffer and incubated with the membrane pieces overnight at 4 °C. The dilution ratios were 1:2,500 for GAPDH, 1:1,000 for phospho-STAT1 (Tyr701), 1:1,000 for total STAT1, and 1:5,000 for the DYKDDDDK tag. After primary-antibody incubation, the membranes were washed three times with 1× TBST for 5 min each and incubated with the corresponding HRP-conjugated secondary antibodies at a dilution of 1:5,000 for 1 h at room temperature. The membranes were then washed three times with 1× TBST for 5 min each.

For sequential detection, the high-molecular-weight membrane strip was first probed for phospho-STAT1 (Tyr701). After chemiluminescence detection, the membrane strip was heated in a boiling water bath for 10 min and directly reprobed for total STAT1 without additional blocking. GAPDH was detected separately using the corresponding low-molecular-weight membrane strip. Immunoreactive bands were visualized using Super ECL Detection Reagent and captured using a Tanon-4600 imaging system. All immunoblotting experiments were independently repeated three times. Representative immunoblots are shown without densitometric quantification.

### 2.18 RNA sequencing and exploratory transcriptomic analysis

Tb 1 Lu cells were transfected with either the empty vector or pcDNA3.1-TBIFN-β-Flag. Total RNA was collected at 24 h after transfection, with three independent biological replicates prepared for each group. RNA sequencing and primary bioinformatic analyses were performed by Sangon Biotech (Shanghai) Co., Ltd. Raw reads were assessed using FastQC version 0.11.2 and filtered using fastp version 0.23.4 to remove adapter sequences, low-quality reads, and reads containing ambiguous bases. Clean reads were assembled *de novo* using Trinity version 2.4.0. Transcript abundance was quantified using Salmon version 0.8.2 and expressed as transcripts per million. Differential expression analysis between the TBIFN-β-overexpression and empty-vector groups was performed using DESeq2 version 1.46.0. Differentially expressed genes were identified using an adjusted P value < 0.05 and an absolute log_2_ fold change > 0.585 as the significance thresholds.

Gene Ontology (GO) enrichment analysis was performed for all differentially expressed genes using topGO version 2.58.0 and GO.db version 3.20.0. Kyoto Encyclopedia of Genes and Genomes (KEGG) pathway enrichment analysis was performed separately for the upregulated and downregulated gene sets using clusterProfiler version 4.14.4. All genes detected in the RNA-sequencing dataset were used as the background gene set. Enrichment was evaluated using P values, and adjusted P values were also calculated to account for multiple testing. Enrichment bubble plots were generated using the Wekemo Bioinformatics Cloud platform. The Gene Ratio represents the number of differentially expressed genes assigned to a given term or pathway divided by the total number of differentially expressed genes included in the corresponding enrichment analysis. Bubble size represents the gene count, and bubble color represents −log_10_(P value). Selected interferon-stimulated and immune-related genes were further validated by RT-qPCR.

### 2.19 Statistical analysis

Statistical analyses were performed using GraphPad Prism version 9.1.0. Unless otherwise indicated, quantitative cell-based experiments were independently repeated three times, and each independent experiment was treated as one biological replicate (n = 3). For RT-qPCR analysis, each biological sample was analyzed in three technical replicate reactions. For dual-luciferase assays, each condition within an independent experiment was analyzed in three technical replicate wells. Technical replicates were averaged before statistical analysis. Data are presented as the mean ± standard deviation (SD), and error bars represent SD. Comparisons between two groups were performed using a two-tailed unpaired Student’s *t*-test. Comparisons involving one experimental factor and more than two groups were performed using one-way analysis of variance followed by Dunnett’s multiple-comparisons test when the experimental groups were compared with a defined control group. Experiments involving two independent factors were analyzed using two-way analysis of variance, followed by Sidak’s multiple-comparisons test. Relative transcript abundance was displayed as 2^−ΔΔCt, whereas statistical analyses of relative RT-qPCR data were performed using the corresponding ΔCt values. A P value < 0.05 was considered statistically significant. Statistical analysis of the RNA-sequencing data was performed separately as described in Section 2.18. Statistical significance is indicated as follows: ns, not significant; *P < 0.05; **P < 0.01; and ***P < 0.001.

## 3 Results

### 3.1 VSV-GFP and VACV-GFP induce distinct temporal patterns of endogenous TBIFNB and ISG expression in Tb 1 Lu cells

**Figure 1:**
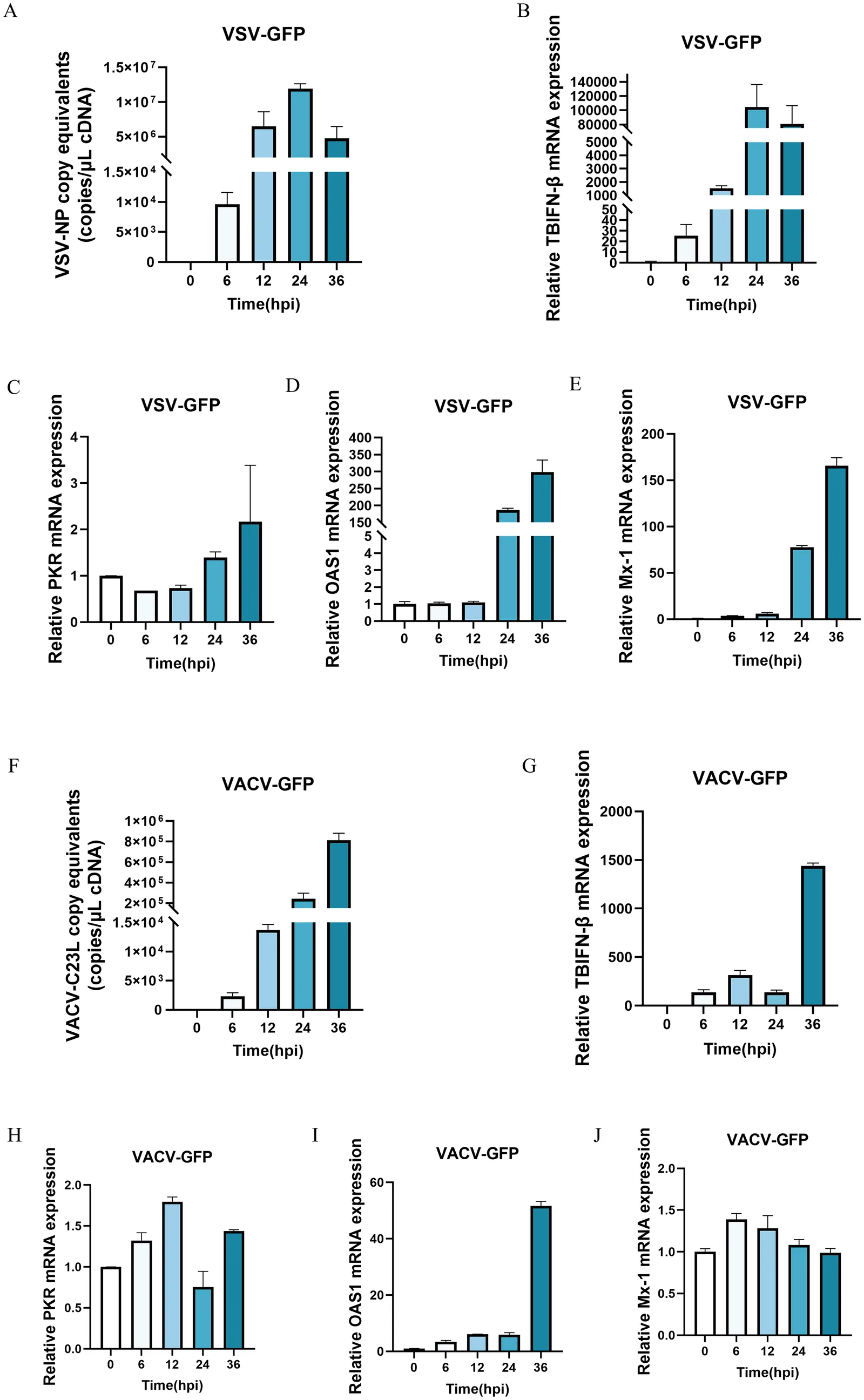
Temporal expression patterns of viral transcripts, TBIFNB, and interferon-stimulated genes in VSV-GFP- and VACV-GFP-infected Tb 1 Lu cells. Tb 1 Lu cells were infected with VSV-GFP at a multiplicity of infection (MOI) of 0.001 or VACV-GFP at an MOI of 0.1 and collected at 0, 6, 12, 24, and 36 h post-infection (hpi). **(A–E)** VSV-NP transcript copy equivalents **(A)** and relative mRNA levels of TBIFNB **(B)**, PKR **(C)**, OAS1 **(D)**, and Mx1 **(E)** following VSV-GFP infection. **(F–J)** VACV-C23L transcript copy equivalents **(F)** and relative mRNA levels of TBIFNB **(G)**, PKR **(H)**, OAS1 **(I)**, and Mx1 **(J)** following VACV-GFP infection. VSV-NP and VACV-C23L transcript copy equivalents were calculated using the corresponding external standard curves and expressed as copies per microliter of the original cDNA preparation without normalization to β-actin. Host-gene transcript levels were normalized to β-actin and calculated using the 2^−ΔΔCt method, with the corresponding 0-h group used as the calibrator. Data are presented as the mean ± SD from three independent experiments (n = 3), and error bars represent SD.

To characterize the innate immune responses of Tb 1 Lu cells to RNA and DNA virus infection, cells were infected with VSV-GFP or VACV-GFP and monitored for up to 36 h. GFP fluorescence became detectable at 12 h post-infection (hpi) and increased progressively thereafter. VSV-GFP spread rapidly across the cell monolayer, whereas VACV-GFP showed a comparatively slower increase in fluorescence, followed by extensive infection at 36 hpi (**Supplementary Figures 1A, B**). Consistent with these observations, RT-qPCR showed progressive accumulation of VSV-NP and VACV-C23L transcripts, with the highest levels detected at 36 hpi (**Figures 1A, F**).

VSV-GFP infection strongly induced endogenous TBIFNB expression. TBIFNB mRNA increased from 6 hpi, rose markedly between 12 and 24 hpi, and remained elevated at 36 hpi (**Figure 1B**). The downstream ISGs exhibited distinct temporal responses. PKR expression decreased slightly during the early stage of infection and subsequently increased at 24 and 36 hpi (**Figure 1C**). In contrast, OAS1 and Mx1 showed pronounced delayed induction, with substantial increases detected at 24 and 36 hpi (**Figures 1D, E**).

VACV-GFP infection produced a different transcriptional response. TBIFNB expression increased at 6 and 12 hpi, declined at 24 hpi, and reached its highest level at 36 hpi (**Figure 1G**). PKR expression fluctuated modestly, showing a transient increase at 12 hpi, a decrease at 24 hpi, and a partial recovery at 36 hpi (**Figure 1H**). OAS1 expression increased moderately during the early phase of infection and was strongly induced at 36 hpi (**Figure 1I**). In contrast, Mx1 expression remained close to the basal level throughout the examined period (**Figure 1J**).

Together, these results show that both VSV-GFP and VACV-GFP established progressive infection in Tb 1 Lu cells and induced endogenous TBIFNB expression. However, the downstream ISG profiles differed markedly between the two viruses. VSV-GFP induced strong late-stage expression of both OAS1 and Mx1, whereas the response to VACV-GFP was more restricted and was characterized predominantly by late OAS1 induction.

### 3.2 Evolutionary conservation, sequence variation, and predicted structure of TBIFN-β

**Figure 2:**
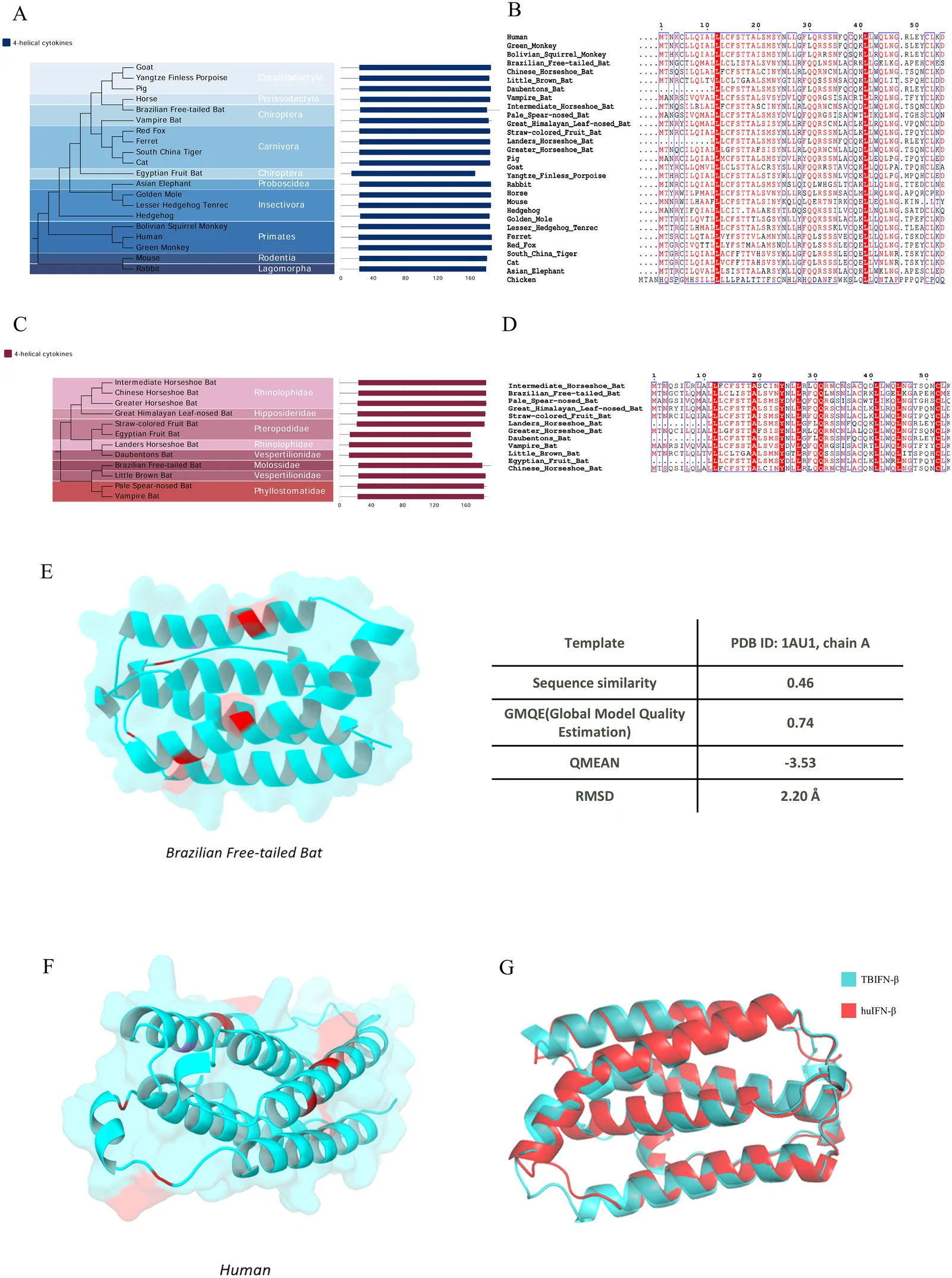
Phylogenetic, sequence, and structural characterization of TBIFN-β. **(A)** Maximum-likelihood phylogenetic tree constructed using IFN-β amino acid sequences from representative mammalian species, with chicken included as an outgroup. Taxonomic orders are indicated by background shading. InterPro-annotated four-helical cytokine domains are shown to the right of the tree, with the scale indicating amino acid position. **(B)** N-terminal multiple sequence alignment of representative mammalian IFN-β proteins. The highlighted column indicates residue 48, numbered according to the full-length precursor protein. Alanine was present at this position in *Tadarida brasiliensis*, horse, and Asian elephant among the species examined. **(C)** Maximum-likelihood phylogenetic tree constructed using IFN-β amino acid sequences from representative bat species. Bat families are indicated by background shading, and the corresponding four-helical cytokine domains are shown to the right. **(D)** N-terminal multiple sequence alignment of bat IFN-β proteins showing sequence variation at residue 48. *T. brasiliensis* contained alanine at this position, whereas the other bat sequences examined contained different residues. **(E)** Predicted three-dimensional structure of TBIFN-β generated by homology modeling using SWISS-MODEL, with human IFN-β (PDB ID: 1AU1, chain A) as the structural template. Model parameters, including sequence similarity, Global Model Quality Estimate (GMQE), QMEAN score, and root-mean-square deviation (RMSD), are shown in the accompanying table. **(F)** Experimentally determined three-dimensional structure of human IFN-β retrieved from the Protein Data Bank (PDB ID: 1AU1, chain A). **(G)** Structural superimposition of the predicted TBIFN-β model and human IFN-β. TBIFN-β is shown in cyan and human IFN-β in red. The Cα RMSD between the aligned structures was 2.20 Å. Phylogenetic trees were constructed using the maximum-likelihood method with the JTT substitution model and 1,000 bootstrap replicates.

To characterize the evolutionary and structural features of TBIFN-β, IFN-β amino acid sequences from representative mammalian species were subjected to phylogenetic and comparative sequence analyses. The mammalian phylogenetic tree showed substantial sequence divergence among taxonomic orders while retaining broadly comparable protein lengths across most species examined (**Figure 2A**). A separate analysis of chiropteran IFN-β sequences showed additional sequence diversity among bat families, with *Tadarida brasiliensis* positioned within the Molossidae lineage (**Figure 2C**). Pairwise comparison of the full-length amino acid sequences further quantified the degree of sequence conservation. TBIFN-β shared 68.1%, 64.5%, 62.4%, and 57.3% amino acid identity with IFN-β from the straw-colored fruit bat, little brown bat, Chinese horseshoe bat, and common vampire bat, respectively. Among representative non-bat mammals, TBIFN-β shared 64.5% identity with pig, 62.9% with horse, 55.4% with human, and 43.1% with mouse IFN-β.

Multiple sequence alignment revealed that many residues were conserved across mammalian IFN-β proteins, although several variable positions were observed, particularly within the N-terminal region (**Figure 2B**; **Supplementary Figure 2A**). Residue 48, numbered according to the full-length precursor protein, showed considerable interspecies variation. Threonine and arginine were frequently observed at this position, whereas alanine was present in *Tadarida brasiliensis*, horse, and Asian elephant among the species included in the present analysis (**Figures 2B, D**; **Supplementary Figures 2A, B**). The identity of alanine at residue 48 in TBIFN-β was further supported by the transcriptome-derived sequence. A separate analysis was performed using representative IFN-β sequences from multiple bat families. Detailed sequence information is provided in **Supplementary Table 2**.

The three-dimensional structure of TBIFN-β was subsequently modeled using human IFN-β (PDB ID: 1AU1, chain A) as the structural template. The resulting model had a template sequence similarity of 0.46, a GMQE value of 0.74, and a QMEAN score of −3.53 (**Figure 2E**). The predicted TBIFN-β structure retained the characteristic α-helical cytokine architecture and four-helix bundle core observed in type I interferons.

Comparison with the experimentally determined human IFN-β structure showed substantial overlap between the two proteins (**Figures 2F, G**). Structural superimposition yielded a root-mean-square deviation of 2.20 Å, indicating that the overall core fold was broadly conserved despite considerable sequence divergence.

Together, these analyses indicate that TBIFN-β retains the overall structural framework characteristic of mammalian IFN-β while containing species-associated sequence variation. Alanine at residue 48 was shared with horse and Asian elephant but differed from the residues present in most of the other species examined. Structural modeling alone does not establish whether variation at this position affects receptor binding or downstream biological activity, which requires direct functional evaluation.

### 3.3 Construction and functional validation of a TBIFNB promoter reporter system

**Figure 3:**
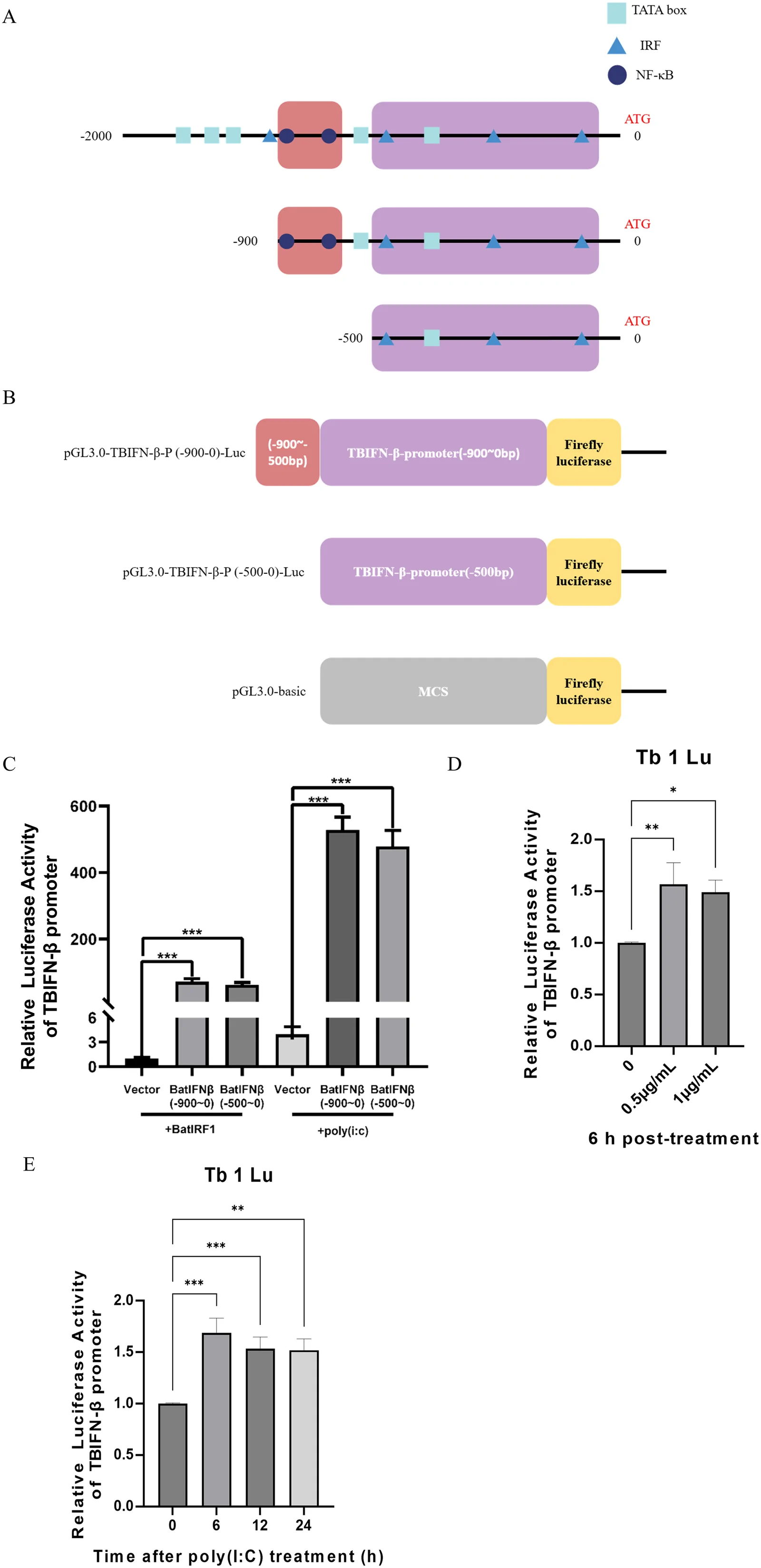
Construction and functional characterization of the TBIFNB promoter reporter system. **(A)** Schematic representation of the approximately 2,000-bp upstream region of TBIFNB and the −900/0 and −500/0 promoter fragments. Nucleotide positions are indicated relative to the translation start site, with the ATG codon defined as position 0. Predicted TATA boxes and putative binding sites for interferon regulatory factors (IRFs) and NF-κB are indicated. **(B)** Schematic representation of the −900/0 and −500/0 TBIFNB promoter reporter constructs. The indicated promoter fragments were inserted upstream of the firefly luciferase gene in the pGL3.0-basic vector. pGL3.0-basic was used as the promoterless reporter control. **(C)** Functional evaluation of the −900/0 and −500/0 promoter constructs following batIRF1 expression or poly(I:C) treatment in Tb 1 Lu cells. Cells were co-transfected with the indicated firefly luciferase reporter construct and pRL-TK. For batIRF1 stimulation, cells additionally received the batIRF1 expression plasmid. For poly(I:C) stimulation, poly(I:C) was added directly to the culture medium. Firefly luciferase activity was normalized to Renilla luciferase activity. **(D)** TBIFNB promoter reporter activity in Tb 1 Lu cells treated with 0, 0.5, or 1 μg/mL poly(I:C) for 6 h. **(E)** Time-dependent TBIFNB promoter reporter activity in Tb 1 Lu cells treated with 1 μg/mL poly(I:C) for 0, 6, 12, or 24 h. For panels **(D, E)**, reporter activity was normalized to that of the corresponding untreated control, which was set to 1. Data are presented as the mean ± SD from three independent experiments (n = 3), each performed with three technical replicate wells. Technical replicates were averaged within each independent experiment before statistical analysis, and error bars represent SD. Statistical significance was assessed using one-way ANOVA followed by Dunnett’s multiple-comparisons test, with the indicated treatment groups compared with the corresponding control group. *P < 0.05; **P < 0.01; ***P < 0.001.

An approximately 2,000-bp genomic region upstream of the TBIFNB translation start site was amplified from Tb 1 Lu cells, and the identity of the amplified sequence was confirmed by sequencing (**Supplementary Figures 3A, B**). Bioinformatic analysis identified multiple predicted TATA boxes and putative binding sites for IRF and NF-κB transcription factors within this region. These predicted regulatory elements were mainly distributed within the −900/0 region, with several predicted IRF-binding sites retained within the proximal −500/0 region (**Figure 3A**).

Based on the predicted distribution of regulatory elements, two promoter fragments spanning −900/0 and −500/0 relative to the translation start site were generated. Both fragments were amplified at the expected sizes (**Supplementary Figure 3C**) and inserted upstream of the firefly luciferase gene in the pGL3.0-basic vector, generating pGL3.0-TBIFN-β-P(−900/0)-Luc and pGL3.0-TBIFN-β-P(−500/0)-Luc, respectively (**Figure 3B**).

The functional responsiveness of the two promoter constructs was evaluated in Tb 1 Lu cells using a dual-luciferase reporter assay. In the presence of batIRF1, both the −900/0 and −500/0 constructs showed substantially higher relative luciferase activity than the pGL3.0-basic control. Both constructs also exhibited marked reporter activity following poly(I:C) treatment (**Figure 3C**). The −500/0 fragment retained a response comparable to that of the longer −900/0 fragment under both experimental conditions, suggesting that regulatory elements sufficient to support promoter activation are located within the proximal 500-bp region.

The response of the TBIFNB promoter reporter to different poly(I:C) conditions was subsequently examined in Tb 1 Lu cells. At 6 h after treatment, both 0.5 and 1 μg/mL poly(I:C) significantly increased relative promoter activity compared with the untreated control. Increasing the concentration from 0.5 to 1 μg/mL did not further increase the reporter response under this condition (**Figure 3D**). Time-course analysis using 1 μg/mL poly(I:C) showed significant promoter activation at 6, 12, and 24 h after treatment. The highest observed response occurred at 6 h and remained elevated, although at a slightly lower level, at 12 and 24 h (**Figure 3E**).

To provide descriptive benchmarking across different IFNB promoter reporter systems, parallel poly(I:C) stimulation experiments were performed using huIFNB promoter reporters in HEK293T and HeLa cells and the TBIFNB promoter reporter in Tb 1 Lu cells. In HEK293T cells, 1 μg/mL poly(I:C) produced marked reporter activation at 6, 12, and 24 h, whereas the response to 0.5 μg/mL reached significance only at 6 h (**Supplementary Figure 4A**). In HeLa cells, both poly(I:C) concentrations significantly activated the huIFNB promoter reporter at all three time points, with relatively stable responses from 6 to 24 h (**Supplementary Figure 4B**). In Tb 1 Lu cells, both concentrations significantly increased TBIFNB promoter activity at 6 and 12 h. At 24 h, significant activation was maintained following treatment with 1 μg/mL poly(I:C), whereas the response to 0.5 μg/mL was no longer significant (**Supplementary Figure 4C**).

The human-cell reporter assays showed larger observed induction amplitudes than the TBIFNB reporter assay under the tested conditions. However, because different cell lines and species-specific promoter constructs were used, these results were considered descriptive and were not interpreted as a direct comparison of intrinsic promoter sensitivity.

Collectively, these results demonstrate that the cloned proximal TBIFNB promoter responds to both batIRF1 expression and poly(I:C) treatment in Tb 1 Lu cells. The −500/0 fragment retained substantial reporter responsiveness, supporting its use as a functional reporter for monitoring TBIFNB promoter activation under the experimental conditions tested. The parallel human-cell assays further showed that the magnitude and temporal pattern of poly(I:C)-induced IFNB promoter activation varied according to the cellular and promoter context.

### 3.4 TBIFN-β restricts VSV-GFP and VACV-GFP infection in Tb 1 Lu cells

To evaluate the antiviral activity of TBIFN-β, its coding sequence was amplified from Tb 1 Lu cell-derived cDNA, yielding a single PCR product of the expected size. The amplified sequence was subsequently cloned into the pcDNA3.1(+) vector to generate the C-terminally Flag-tagged expression plasmid pcDNA3.1-TBIFN-β-Flag (**Supplementary Figures 5A, B**).

**Figure 4:**
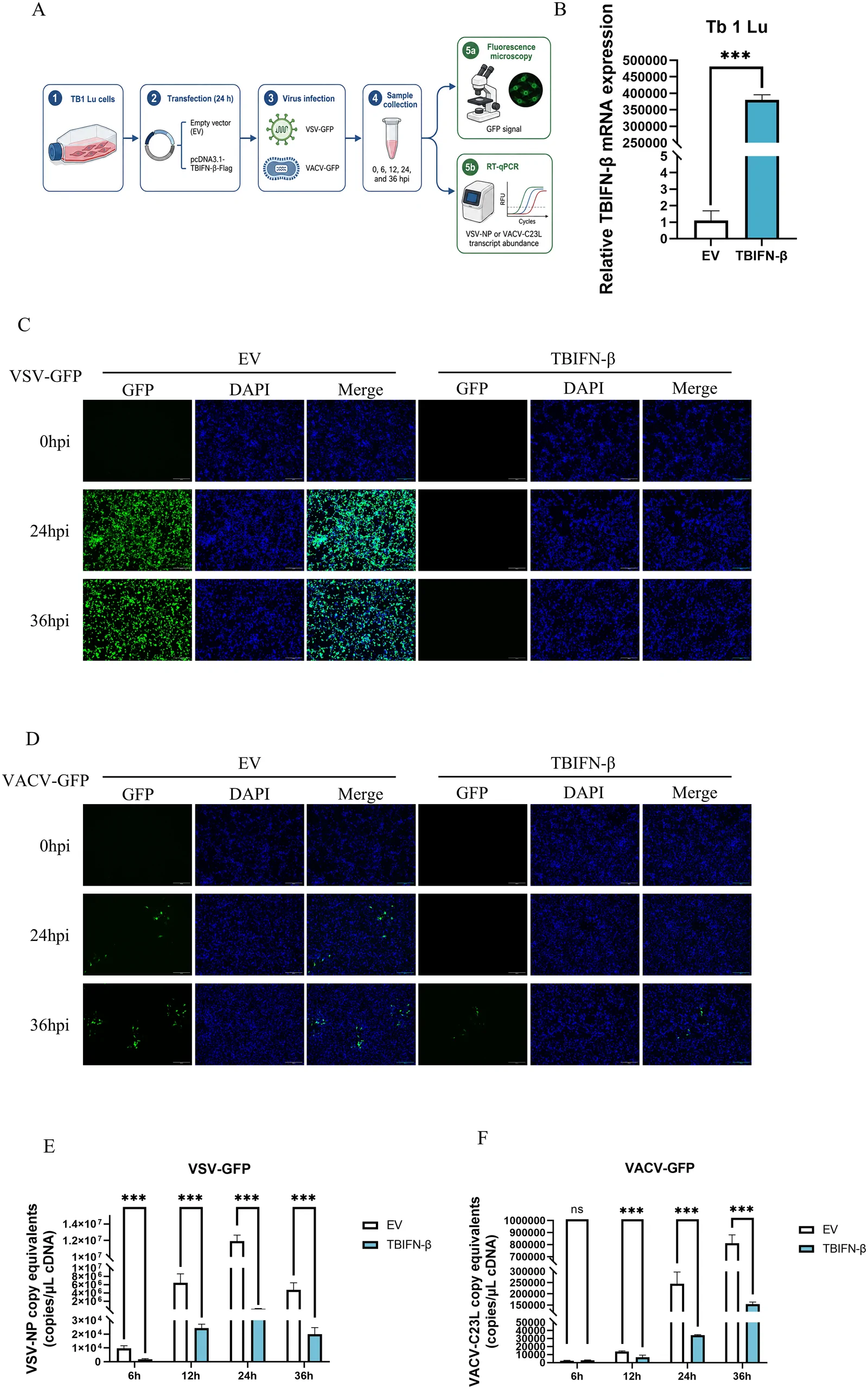
Transfection with the TBIFN-β expression construct restricts VSV-GFP and VACV-GFP infection in Tb 1 Lu cells. **(A)** Schematic representation of the experimental workflow. Tb 1 Lu cells were transfected with either the empty vector (EV) or pcDNA3.1-TBIFN-β-Flag for 24 h and subsequently infected with VSV-GFP or VACV-GFP. Samples were collected at the indicated time points for fluorescence microscopy and RT-qPCR analysis. **(B)** Relative TBIFNB mRNA levels in Tb 1 Lu cells at 24 h after transfection with EV or pcDNA3.1-TBIFN-β-Flag. TBIFNB transcript levels were normalized to β-actin and calculated using the 2^−ΔΔCt method, with the EV group used as the calibrator. **(C)** Representative fluorescence microscopy images showing GFP fluorescence, DAPI nuclear staining, and merged channels in VSV-GFP-infected cells transfected with EV or pcDNA3.1-TBIFN-β-Flag at 0, 24, and 36 h post-infection (hpi). **(D)** Representative fluorescence microscopy images showing GFP fluorescence, DAPI nuclear staining, and merged channels in VACV-GFP-infected cells transfected with EV or pcDNA3.1-TBIFN-β-Flag at 0, 24, and 36 hpi. **(E)** VSV-NP transcript copy equivalents in EV- and pcDNA3.1-TBIFN-β-Flag-transfected cells at 6, 12, 24, and 36 hpi. **(F)** VACV-C23L transcript copy equivalents in EV- and pcDNA3.1-TBIFN-β-Flag-transfected cells at 6, 12, 24, and 36 hpi. GFP fluorescence images in panels **(C, D)** were acquired using a 10× objective under fixed acquisition settings for the corresponding experimental comparisons. GFP fluorescence was used for qualitative visualization of viral infection and was not subjected to quantitative fluorescence-intensity analysis. VSV-NP and VACV-C23L transcript copy equivalents were calculated using the corresponding external standard curves and expressed as copies per microliter of the original cDNA preparation without normalization to β-actin. Data are presented as the mean ± SD from three independent experiments (n = 3), and error bars represent SD. The fluorescence images are representative of three independent experiments. Panel B was analyzed using a two-tailed unpaired Student’s t-test. Panels **(E, F)** were analyzed using two-way ANOVA followed by Sidak’s multiple-comparisons test. ns, not significant; ***P < 0.001. Scale bars in panels C and D, 200 μm.

**Figure 5:**
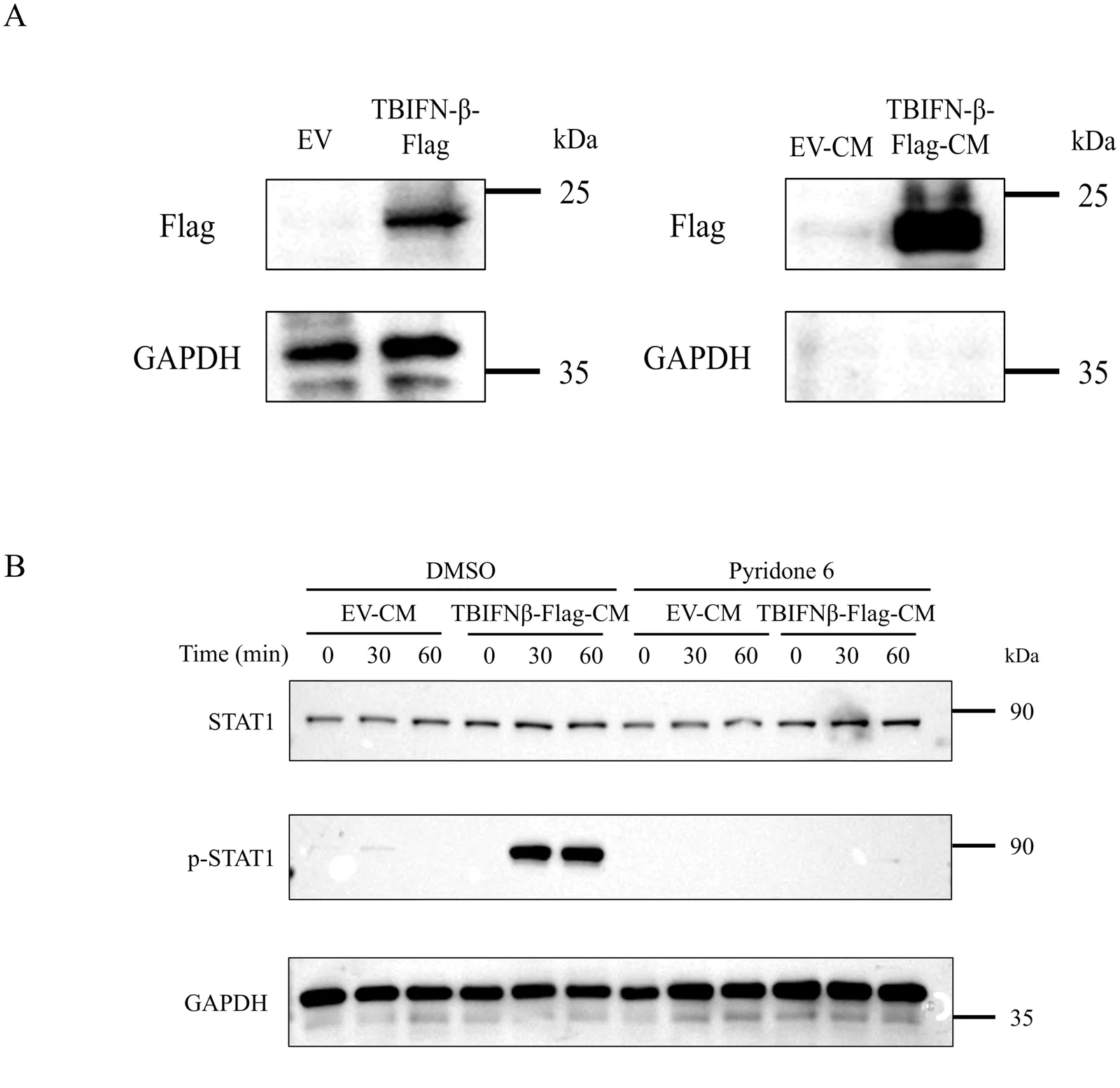
Detection of Flag-tagged TBIFN-β in conditioned medium and Pyridone 6-sensitive STAT1 phosphorylation in recipient cells. **(A)** Immunoblot detection of Flag-tagged TBIFN-β in cellular lysates and concentrated conditioned-medium samples from Tb 1 Lu cells transfected with the empty vector (EV) or pcDNA3.1-TBIFN-β-Flag. GAPDH was examined in both cellular lysates and conditioned-medium samples as an indicator of cellular protein contamination. EV-CM, conditioned medium collected from EV-transfected cells; TBIFN-β-Flag-CM, conditioned medium collected from pcDNA3.1-TBIFN-β-Flag-transfected cells. **(B)** Recipient Tb 1 Lu cells were pretreated with DMSO or 10 μM Pyridone 6 for 2 h and subsequently exposed to EV-CM or TBIFN-β-Flag-CM. Cells were collected at 0, 30, and 60 min after conditioned-medium addition, and total STAT1, phospho-STAT1 (Tyr701), and GAPDH were detected by immunoblotting. The 0-min samples were collected immediately after conditioned-medium addition. Representative immunoblots from three independent experiments are shown. No densitometric quantification was performed.

Tb 1 Lu cells were transfected with either pcDNA3.1-TBIFN-β-Flag or the corresponding empty vector for 24 h and were subsequently infected with VSV-GFP or VACV-GFP. Viral infection was visualized qualitatively by GFP fluorescence microscopy, whereas viral transcript abundance was quantified by RT-qPCR at the indicated time points (**Figure 4A**). RT-qPCR confirmed a marked increase in TBIFNB mRNA in cells transfected with pcDNA3.1-TBIFN-β-Flag compared with the empty-vector group, demonstrating efficient transcriptional overexpression of TBIFN-β (**Figure 4B**). Protein-level expression and extracellular detection of Flag-tagged TBIFN-β were subsequently confirmed by immunoblotting (**Figure 5A**).

Following VSV-GFP infection, GFP fluorescence progressively increased in empty-vector-transfected cells, becoming readily detectable at 12 h post-infection (hpi) and spreading extensively across the cell monolayer at 24 and 36 hpi. In contrast, GFP-positive cells remained sparse in the TBIFN-β-transfected group throughout the examined period (**Figure 4C**). The complete fluorescence time course at both 4× and 10× magnification further showed a marked reduction in VSV-GFP fluorescence in TBIFN-β-transfected cells at 12, 24, and 36 hpi (**Supplementary Figure 5C**). Consistent with the fluorescence observations, VSV-NP transcript copy equivalents were significantly lower in the TBIFN-β-transfected group than in the empty-vector group at 6, 12, 24, and 36 hpi (**Figure 4E**).

A similar inhibitory effect was observed following VACV-GFP infection. In empty-vector-transfected cells, GFP-positive foci became evident at 12 hpi and increased substantially at 24 and 36 hpi. By comparison, VACV-GFP fluorescence remained markedly reduced in cells transfected with the TBIFN-β expression construct (**Figure 4D**). The complete 4× and 10× fluorescence series confirmed reduced VACV-GFP spread in the TBIFN-β-transfected group throughout the later stages of infection (**Supplementary Figure 5D**). RT-qPCR showed no significant difference in VACV-C23L transcript copy equivalents between the two groups at 6 hpi. However, VACV-C23L transcript copy equivalents were significantly lower in TBIFN-β-transfected cells at 12, 24, and 36 hpi (**Figure 4F**).

Collectively, these results show that transfection with the TBIFN-β expression construct restricted infection by both VSV-GFP and VACV-GFP in Tb 1 Lu cells. The inhibitory effect against VSV-GFP was detectable throughout the examined infection period, whereas the reduction in VACV-GFP target transcript abundance became evident from 12 hpi.

### 3.5 TBIFN-β preferentially activates OAS1 and induces virus-dependent ISG responses

To characterize the downstream antiviral response induced by TBIFN-β, the expression of three representative interferon-stimulated genes, PKR, OAS1, and Mx1, was examined in Tb 1 Lu cells transfected with either the empty vector or pcDNA3.1-TBIFN-β-Flag. Cells were analyzed under mock conditions or following infection with VSV-GFP, VACV-GFP, PRV-GFP, or H1N1.

**Figure 6:**
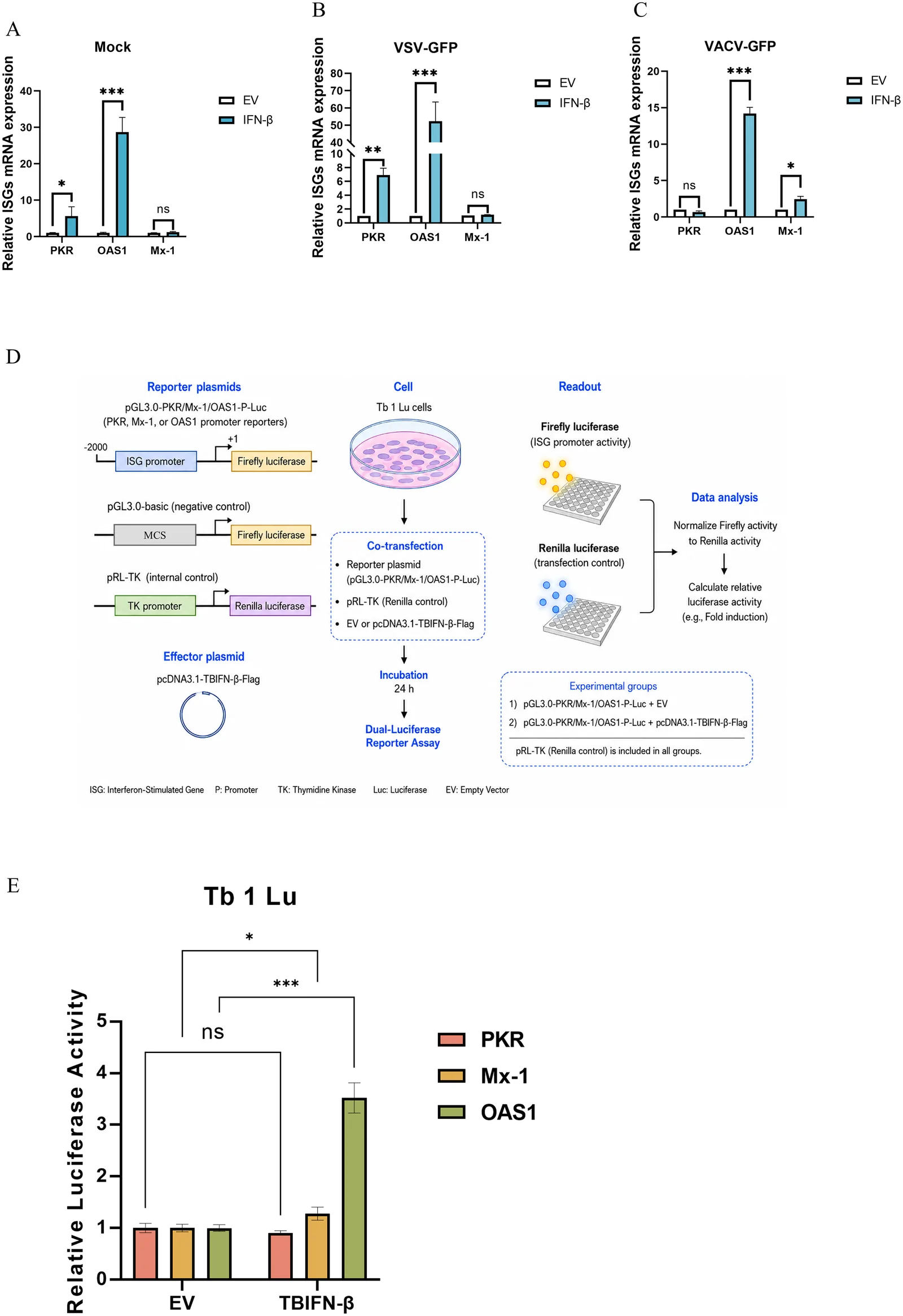
Differential regulation of endogenous ISG expression and ISG promoter activity following transfection with the TBIFN-β expression construct. Tb 1 Lu cells were transfected with either the empty vector (EV) or pcDNA3.1-TBIFN-β-Flag for 24 h and were subsequently mock infected or infected with VSV-GFP or VACV-GFP. Samples were collected at 24 h post-infection for RT-qPCR analysis. **(A–C)** Relative mRNA levels of PKR, OAS1, and Mx1 in EV- and pcDNA3.1-TBIFN-β-Flag-transfected cells under mock conditions **(A)**, following VSV-GFP infection **(B)**, or following VACV-GFP infection **(C)**. Transcript levels were normalized to β-actin and calculated using the 2^−ΔΔCt method, with the corresponding EV group used as the calibrator. **(D)** Schematic representation of the ISG promoter reporter assay. Tb 1 Lu cells were co-transfected with the indicated PKR, Mx1, or OAS1 promoter firefly luciferase reporter construct, pRL-TK, and either EV or pcDNA3.1-TBIFN-β-Flag. Dual-luciferase activity was measured after 24 h. **(E)** Relative activities of the PKR, Mx1, and OAS1 promoter reporters in EV- and pcDNA3.1-TBIFN-β-Flag-transfected cells. Firefly luciferase activity was normalized to Renilla luciferase activity, and the activity of each corresponding EV group was set to 1. Data are presented as the mean ± SD from three independent experiments (n = 3), and error bars represent SD. For the dual-luciferase reporter assay, each independent experiment was performed with three technical replicate wells, which were averaged before statistical analysis. Panels **(A–C)** were analyzed using two-way ANOVA followed by Sidak’s multiple-comparisons test. Panel **(E)** was analyzed using two-way ANOVA followed by Sidak’s multiple-comparisons test; ns, not significant; *P < 0.05; **P < 0.01; ***P < 0.001.

Under mock conditions, transfection with the TBIFN-β expression construct significantly increased PKR and OAS1 mRNA levels compared with the empty-vector control. OAS1 showed the strongest response, reaching approximately 28-fold relative to the control, whereas Mx1 expression was not significantly altered (**Figure 6A**).

Following VSV-GFP infection, TBIFN-β significantly increased both PKR and OAS1 expression. PKR mRNA increased by approximately sevenfold, whereas OAS1 reached approximately 50-fold relative to the corresponding empty-vector group. In contrast, Mx1 expression was not significantly affected (**Figure 6B**).

A different ISG expression profile was observed following VACV-GFP infection. PKR expression did not differ significantly between the TBIFN-β and empty-vector groups. OAS1 expression was markedly increased, reaching approximately 14-fold relative to the control, whereas Mx1 showed a modest but significant increase of approximately twofold (**Figure 6C**).

Additional virus infection experiments further demonstrated that the effects of TBIFN-β varied according to the viral context. Following PRV-GFP infection, none of the three examined ISGs showed a statistically significant change, although their mean expression levels were slightly higher in the TBIFN-β group (**Supplementary Figure 6A**). Following IAV-H1N1 infection, only OAS1 was significantly increased, whereas PKR and Mx1 remained unchanged (**Supplementary Figure 6B**).

To determine whether the differential ISG responses were also reflected at the promoter level, reporter constructs containing the PKR, Mx1, or OAS1 promoter upstream of the firefly luciferase gene were evaluated in Tb 1 Lu cells. Each reporter construct was co-transfected with pRL-TK and either the empty vector or pcDNA3.1-TBIFN-β-Flag, and luciferase activity was measured after 24 h (**Figure 6D**).

TBIFN-β did not significantly alter PKR promoter activity. In contrast, Mx1 promoter activity showed a modest but significant increase, whereas OAS1 promoter activity increased by approximately 3.5-fold and displayed the strongest response among the three reporter constructs (**Figure 6E**).

Collectively, these results demonstrate that TBIFN-β does not uniformly activate all downstream ISGs. OAS1 was consistently induced under mock, VSV-GFP, VACV-GFP, and H1N1 conditions and also showed the strongest promoter-level response. In contrast, PKR and Mx1 displayed weaker and virus-dependent expression patterns. These findings support context-dependent regulation of downstream antiviral responses by TBIFN-β in Tb 1 Lu cells.

### 3.6 Pharmacological inhibition of JAK signaling attenuates TBIFN-β-induced ISG expression and antiviral activity

To assess the contribution of JAK signaling to the downstream effects of TBIFN-β, Tb 1 Lu cells were transfected with either the empty vector or pcDNA3.1-TBIFN-β-Flag. At 24 h after transfection, cells were pretreated with 10 μM Pyridone 6, a JAK inhibitor, or the corresponding DMSO vehicle for 2 h and were subsequently mock infected or infected with VSV-GFP or VACV-GFP. Samples were collected at 24 h post-infection for RT-qPCR analysis, and viral infection was additionally evaluated by GFP fluorescence microscopy.

**Figure 7:**
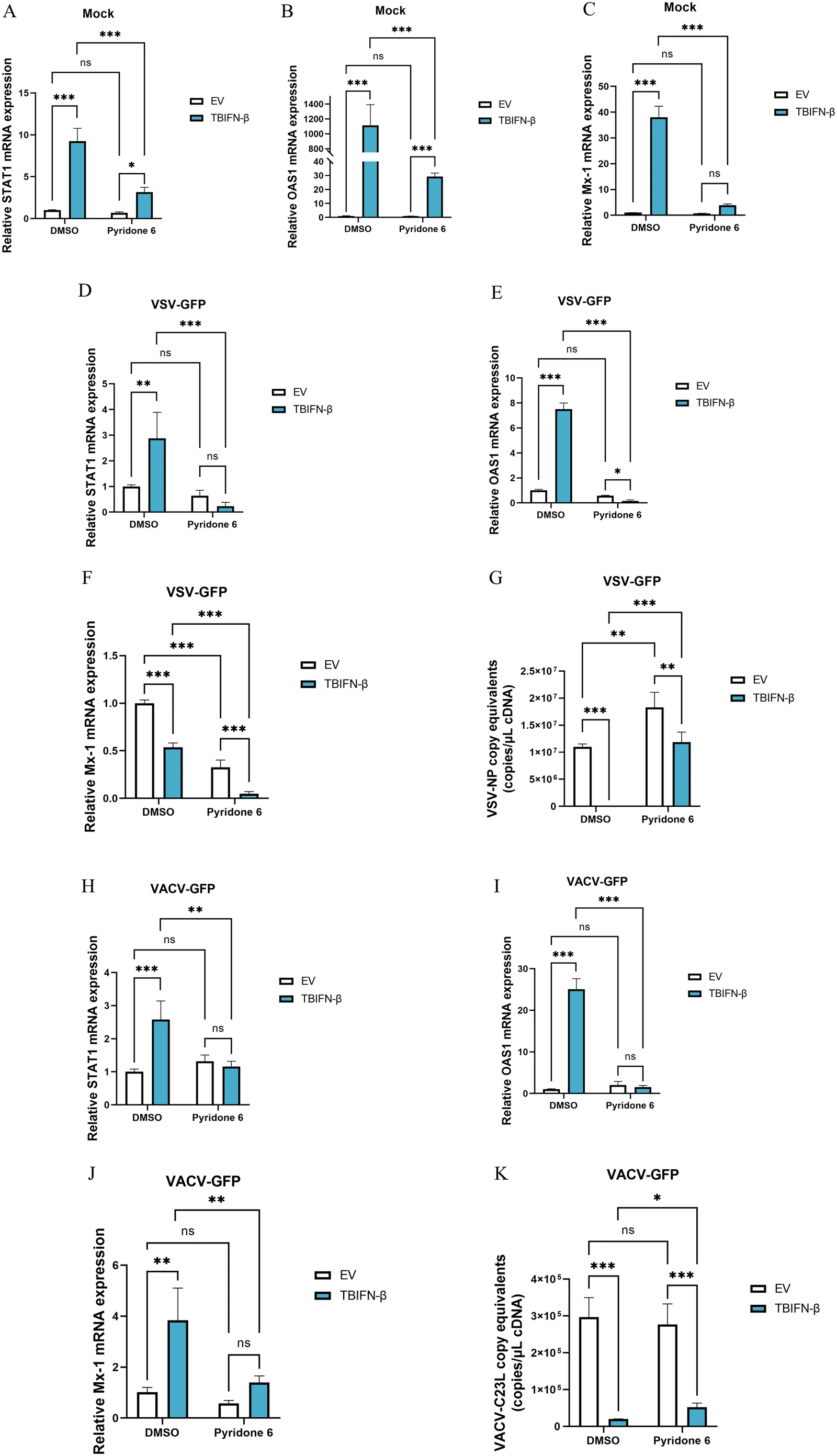
Pyridone 6 attenuates TBIFN-β-induced gene expression and antiviral activity in Tb 1 Lu cells. Tb 1 Lu cells were transfected with either the empty vector (EV) or pcDNA3.1-TBIFN-β-Flag. At 24 h after transfection, cells were pretreated with 10 μM Pyridone 6 or the corresponding DMSO vehicle for 2 h and were subsequently mock infected or infected with VSV-GFP at an MOI of 0.001 or VACV-GFP at an MOI of 0.1. Pyridone 6 or DMSO remained present throughout the infection period, and samples were collected at 24 h post-infection for RT-qPCR analysis. **(A–C)** Relative mRNA levels of STAT1 **(A)**, OAS1 **(B)**, and Mx1 **(C)** under mock conditions. **(D–G)** Relative mRNA levels of STAT1 **(D)**, OAS1 **(E)**, and Mx1 **(F)**, and VSV-NP transcript copy equivalents **(G)**, following VSV-GFP infection. **(H–K)** Relative mRNA levels of STAT1 **(H)**, OAS1 **(I)**, and Mx1 **(J)**, and VACV-C23L transcript copy equivalents **(K)**, following VACV-GFP infection. Host-gene transcript levels were normalized to β-actin and calculated using the 2^−ΔΔCt method, with the EV plus DMSO group used as the calibrator within each infection condition. VSV-NP and VACV-C23L transcript copy equivalents were calculated using the corresponding external standard curves and expressed as copies per microliter of the original cDNA preparation without normalization to β-actin. Data are presented as the mean ± SD from three independent experiments (n = 3), and error bars represent SD. Statistical significance was assessed using two-way ANOVA followed by Sidak’s multiple-comparisons test. ns, not significant; *P < 0.05; **P < 0.01; ***P < 0.001.

Under mock conditions, transfection with the TBIFN-β expression construct markedly increased STAT1, OAS1, and Mx1 mRNA levels in DMSO-treated cells (**Figures 7A–C**). PKR expression was also significantly increased under this condition (**Supplementary Figure 7C**). Pyridone 6 treatment significantly reduced the TBIFN-β-induced expression of all four genes. Nevertheless, STAT1, OAS1, and PKR expression remained significantly higher in TBIFN-β-transfected cells than in their corresponding empty-vector controls, whereas the difference in Mx1 expression was no longer significant.

Following VSV-GFP infection, TBIFN-β increased STAT1 and OAS1 expression in DMSO-treated cells (**Figures 7D, E**; **Supplementary Figure 7D**). Pyridone 6 abolished the increase in STAT1 expression and reduced the magnitude of PKR induction, although PKR expression remained significantly higher in the TBIFN-β group than in the corresponding empty-vector group. In contrast, Mx1 expression was lower in TBIFN-β-transfected cells under both vehicle- and Pyridone 6-treated conditions (**Figure 7F**). Pyridone 6 also reduced Mx1 expression in the empty-vector group, indicating that the inhibitor affected the broader infection-induced signaling response.

TBIFN-β substantially reduced VSV-NP transcript copy equivalents in DMSO-treated cells. VSV-NP levels remained significantly lower in TBIFN-β-transfected cells after Pyridone 6 treatment but were increased relative to those in the corresponding TBIFN-β plus DMSO group (**Figure 7G**). Fluorescence microscopy similarly showed that Pyridone 6 partially increased VSV-GFP fluorescence in TBIFN-β-transfected cells, although the fluorescence signal remained lower than that in the corresponding empty-vector group (**Supplementary Figure 7A**).

Following VACV-GFP infection, TBIFN-β increased STAT1, OAS1, and Mx1 expression in DMSO-treated cells (**Figures 7H–J**). PKR expression was also increased in the DMSO-treated inhibitor experiment (**Supplementary Figure 7E**). Pyridone 6 markedly attenuated these responses, and none of the four genes remained significantly increased in TBIFN-β-transfected cells relative to the corresponding empty-vector controls.

TBIFN-β also significantly reduced VACV-C23L transcript copy equivalents in both DMSO- and Pyridone 6-treated cells. However, VACV-C23L levels were significantly higher in the TBIFN-β plus Pyridone 6 group than in the TBIFN-β plus DMSO group, indicating partial loss of the inhibitory effect following JAK inhibition (**Figure 7K**). Consistently, Pyridone 6 partially restored VACV-GFP fluorescence in TBIFN-β-transfected cells, although viral fluorescence remained lower than that observed in the corresponding empty-vector group (**Supplementary Figure 7B**).

Collectively, these findings show that pharmacological inhibition of JAK signaling substantially attenuated TBIFN-β-induced ISG expression and partially reduced its inhibitory effects against VSV-GFP and VACV-GFP. However, TBIFN-β retained measurable antiviral activity in the presence of Pyridone 6. This residual activity may reflect incomplete pathway inhibition or additional antiviral mechanisms that were not resolved by the present experiment.

### 3.7 Flag-tagged TBIFN-β is detected in conditioned medium and induces Pyridone 6-sensitive STAT1 phosphorylation

To determine whether the TBIFN-β expression construct produced detectable protein, cellular lysates and concentrated conditioned-medium samples were analyzed by immunoblotting. A Flag-reactive band of approximately 25 kDa was detected in cellular lysates from pcDNA3.1-TBIFN-β-Flag-transfected cells but not in empty-vector controls. A corresponding Flag-reactive band was also detected in conditioned medium collected from TBIFN-β-Flag-transfected cells, whereas no corresponding band was observed in EV-CM (**Figure 5A**). GAPDH was detected in the cellular lysates but not in the conditioned-medium samples, consistent with minimal detectable contamination by intracellular proteins.

The ability of the conditioned medium to activate downstream signaling was subsequently examined in recipient Tb 1 Lu cells. Under DMSO treatment, TBIFN-β-Flag-CM induced a clear phospho-STAT1 (Tyr701) signal at 30 and 60 min, whereas little or no phospho-STAT1 signal was detected at 0 min or following EV-CM treatment. Pretreatment with Pyridone 6 markedly attenuated the phospho-STAT1 response induced by TBIFN-β-Flag-CM, while total STAT1 and GAPDH remained detectable across the examined groups (**Figure 5B**).

Together, these results show that Flag-tagged TBIFN-β was detectable in conditioned medium and that TBIFN-β-Flag-CM rapidly induced STAT1 phosphorylation in a Pyridone 6-sensitive manner. The same qualitative pattern was observed in three independent experiments.

### 3.8 Exploratory transcriptomic analysis reveals coordinated antiviral gene regulation following TBIFN-β overexpression

To obtain a broader view of the transcriptional response associated with TBIFN-β overexpression, RNA sequencing was performed on Tb 1 Lu cells transfected with either the empty vector or pcDNA3.1-TBIFN-β-Flag. Differential expression analysis was performed using DESeq2, and genes meeting the adjusted P-value and fold-change criteria specified in the Methods were considered differentially expressed.

**Figure 8:**
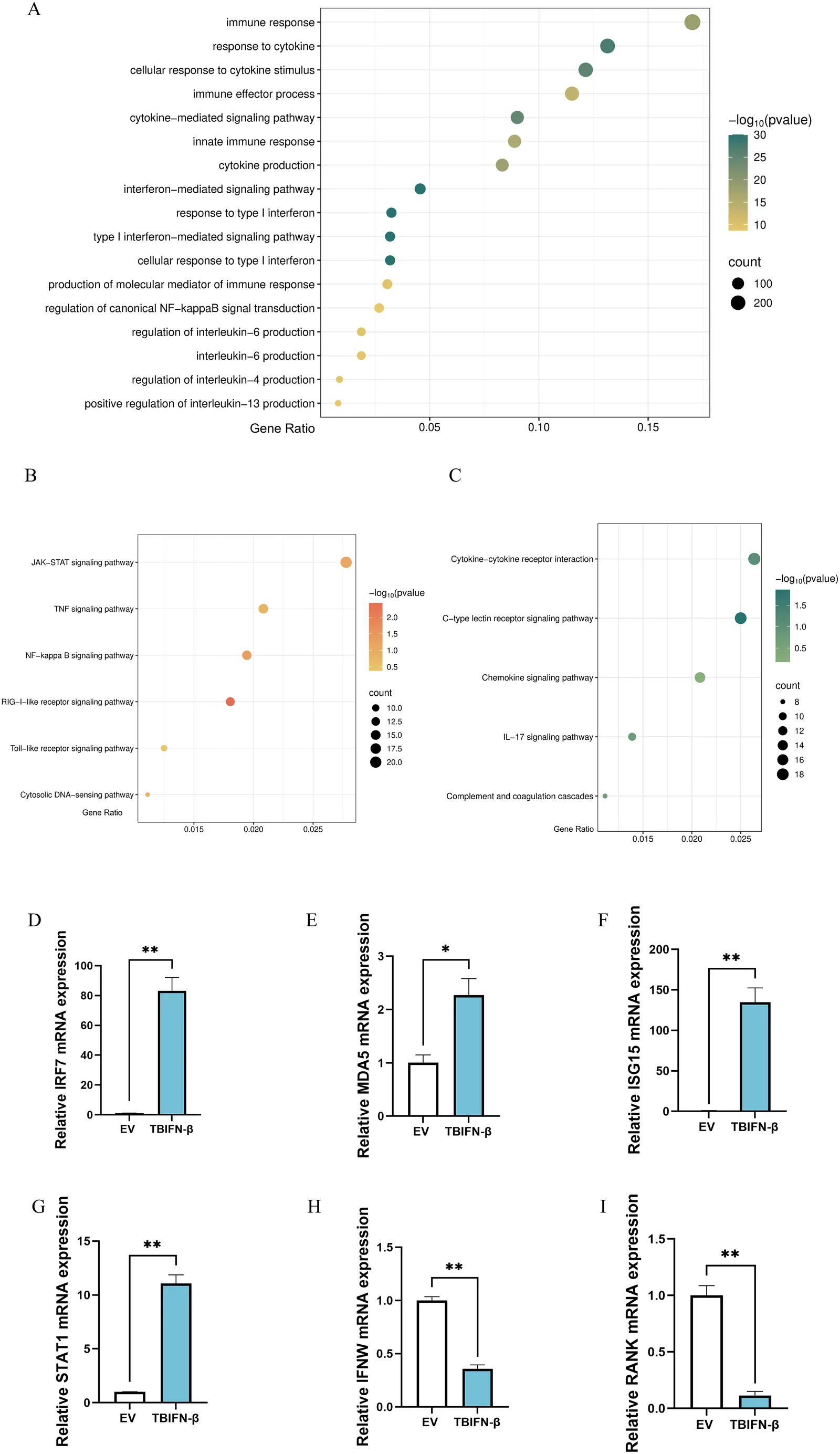
Functional enrichment analysis and RT-qPCR validation of the transcriptional response associated with TBIFN-β overexpression. Tb 1 Lu cells were transfected with either the empty vector (EV) or pcDNA3.1-TBIFN-β-Flag, and total RNA was collected at 24 h after transfection. **(A)** Gene Ontology biological-process enrichment analysis of all differentially expressed genes. **(B)** Kyoto Encyclopedia of Genes and Genomes pathway enrichment analysis of upregulated genes. **(C)** Kyoto Encyclopedia of Genes and Genomes pathway enrichment analysis of downregulated genes. Selected enriched terms and pathways are shown. In panels **(A–C)**, the gene ratio represents the number of differentially expressed genes assigned to a given term or pathway divided by the total number of differentially expressed genes included in the corresponding enrichment analysis. Bubble size represents the gene count, and bubble color represents −log_10_(P value). **(D–G)** Relative mRNA levels of the selected upregulated genes IRF7 **(D)**, MDA5 **(E)**, ISG15 **(F)**, and STAT1 **(G)**. **(H, I)** Relative mRNA levels of the selected downregulated genes IFNW **(H)** and RANK **(I)**. Transcript levels were normalized to β-actin and calculated using the 2^−ΔΔCt method, with the EV group used as the calibrator. Data in panels **(D–I)** are presented as the mean ± SD from three independent experiments (n = 3), and error bars represent SD. Statistical significance was assessed using a two-tailed unpaired Student’s t-test. *P < 0.05; **P < 0.01.

Gene Ontology biological-process enrichment analysis of all differentially expressed genes highlighted immune response, response to cytokine, cellular response to cytokine stimulus, immune effector process, cytokine-mediated signaling, innate immune response, interferon-mediated signaling, response to type I interferon, and type I interferon-mediated signaling among the prominently represented terms (**Figure 8A**).

KEGG enrichment analysis of the upregulated gene set identified the JAK-STAT, TNF, NF-κB, RIG-I-like receptor, Toll-like receptor, and cytosolic DNA-sensing signaling pathways among the displayed enriched pathways (**Figure 8B**). Consistent with these pathway-level findings, 18 selected immune-related transcripts were upregulated following transfection with the TBIFN-β expression construct. These genes included viral sensors and associated regulators, such as MDA5, RIG-I, LGP2, and TLR3; signaling and transcriptional regulators, including IRF7, IRF9, STAT1, STAT2, TRIM25, and SIKE; and antiviral effector genes, including an OAS-family transcript, ISG15, IFIT3, Mx1, GBP2, and RNaseL (**Table 1**). The OAS-family transcript showed the largest increase among the selected genes, with a log_2_ fold change of 9.75. IRF7, MDA5, and ISG15 were also strongly upregulated, with log_2_ fold changes of 6.48, 6.16, and 5.11, respectively.

**Table 1:** Differentially expressed immune-related genes in Tb 1 Lu cells upon TBIFN-β overexpression.

Gene	Description	Log2FC	Adjusted P-value	Regulation
OAS	2’-5’-oligoadenylate synthetase	9.75	1.3E-09	up
IRF7	Interferon regulatory factor 7	6.48	1.6E-49	up
MDA5	Melanoma differentiation-associated gene 5	6.16	2.8E-36	up
ISG15	Interferon-stimulated gene 15	5.11	8.2E-28	up
TRIM25	Tripartite motif containing 25	4.92	7.2E-04	up
SIKE	Suppressor of IKBKE 1	4.12	3.1E-02	up
IFIT3	interferon-induced protein with tetratricopeptide repeats 3	3.60	1.3E-43	up
Mx-1	Myxovirus resistance protein 1	3.25	3.1E-37	up
RIG-I	Retinoic acid-inducible gene-I	2.88	4.2E-28	up
GBP2	Guanylate binding protein 2	2.83	2.8E-20	up
IRF9	Interferon regulatory Factor 9	2.47	1.6E-46	up
STAT1	Signal transducer and activator of transcription 1	2.30	1.0E-31	up
STAT2	Signal transducer and activator of transcription 2	2.15	1.6E-36	up
TLR3	Toll-like receptor 3	1.82	4.5E-03	up
RNaseL	Ribonuclease L	1.39	1.7E-11	up
TREX1	Three prime repair exonuclease 1	1.24	1.6E-12	up
RNF31	Ring finger protein 31	1.24	8.0E-13	up
LGP2(DHX58)	Laboratory of genetics and physiology 2	1.08	2.2E-02	up
IFNW	Interferon omega	-4.22	4.9E-10	down
RANK	Receptor activator of NF-κB	-1.23	7.9E-03	down
cIAP1/2(BIRC2_3)	Baculoviral IAP repeat containing 1/2	-0.86	9.0E-04	down

KEGG enrichment analysis of the downregulated gene set identified cytokine-cytokine receptor interaction, C-type lectin receptor signaling, chemokine signaling, IL-17 signaling, and complement and coagulation cascades among the displayed enriched pathways (**Figure 8C**). Among the selected immune-related transcripts, IFNW showed the largest decrease, with a log_2_ fold change of −4.22, whereas RANK and cIAP1/2 showed more moderate reductions, with log_2_ fold changes of −1.23 and −0.86, respectively (**Table 1**).

RT-qPCR was subsequently used to examine six representative genes selected from the RNA-sequencing dataset. Consistent with the transcriptomic results, IRF7, MDA5, ISG15, and STAT1 mRNA levels were significantly increased in cells transfected with pcDNA3.1-TBIFN-β-Flag compared with empty-vector controls (**Figures 8D–G**). IRF7 and ISG15 showed particularly pronounced induction, whereas MDA5 and STAT1 exhibited more moderate increases. Conversely, IFNW and RANK expression was significantly reduced in the TBIFN-β-transfected group, confirming the direction of change detected by RNA sequencing (**Figures 8H, I**).

Collectively, the GO, KEGG, RNA-sequencing, and RT-qPCR results indicate that TBIFN-β overexpression induces a coordinated transcriptional response involving cytokine and type I interferon responses, viral recognition pathways, JAK-STAT signaling, and downstream antiviral effector genes. Downregulated genes were also associated with several immune-related pathway categories, although these enrichment results alone do not establish functional suppression of the corresponding pathways.

## 4 Discussion

Bats are ecologically diverse mammals with substantial interspecies variation in antiviral immunity, interferon loci, signaling regulators, and antiviral effector genes (2–4, 6, 9). This diversity makes it difficult to generalize findings from one bat species to the entire order Chiroptera. In the present study, we characterized IFN-β from Tadarida brasiliensis at the sequence, predicted structural, promoter, antiviral, protein, signaling, and transcriptomic levels. TBIFN-β retained the predicted structural framework of mammalian IFN-β, and its proximal promoter responded to batIRF1 expression and poly(I:C) treatment. Transfection with the TBIFN-β expression construct was associated with reduced infection by several tested viruses. Flag-tagged TBIFN-β was detected in conditioned medium, and conditioned medium from TBIFN-β-transfected cells induced rapid STAT1 phosphorylation in recipient cells. Downstream transcriptional responses were not uniform, with OAS1 showing the most consistent induction among the three targeted ISGs. Pharmacological inhibition and transcriptomic enrichment analyses further supported the involvement of JAK-dependent interferon signaling. Together, these findings provide a species-specific *in vitro* characterization of TBIFN-β but do not establish a general mechanism of viral tolerance or asymptomatic viral carriage in bats.

VSV-GFP and VACV-GFP both established progressive infection in Tb 1 Lu cells but induced different temporal patterns of endogenous TBIFNB and ISG expression. VSV-GFP induced sustained TBIFNB expression together with pronounced late-stage increases in OAS1 and Mx1. In contrast, VACV-GFP produced a more variable TBIFNB response and a more restricted downstream pattern dominated by late OAS1 induction. These differences may reflect variation in viral replication kinetics, nucleic acid sensing, or viral antagonism of interferon-associated pathways. Vaccinia virus encodes several proteins that interfere with type I interferon responses. B18R acts as a soluble type I interferon-binding protein, whereas E3L and K3L interfere with PKR-associated antiviral activity through distinct mechanisms (10–12). However, the contribution of these viral proteins was not examined here. Their involvement in the responses observed in Tb 1 Lu cells therefore remains speculative.

Structural modeling predicted that TBIFN-β retains the characteristic helical cytokine fold observed in human IFN-β (5, 13). Structural superimposition further indicated broad conservation of the core fold despite considerable sequence divergence. Nevertheless, structural similarity alone cannot demonstrate equivalent receptor binding, signaling potency, or antiviral activity. Residue 48 showed substantial variation among the mammalian IFN-β sequences examined. Alanine was present in *T. brasiliensis*, horse, and Asian elephant, indicating that A48 is neither unique to *T. brasiliensis* nor specific to bats. Nevertheless, its occurrence in *T. brasiliensis* but not in the other selected bat sequences represents an interesting comparative observation. The present sequence analysis and homology model cannot determine whether this residue affects receptor binding, signaling potency, or antiviral activity. Site-directed mutagenesis and direct functional comparisons will be required to establish its biological relevance. Previous mutational mapping of human IFN-β has shown that substitutions at exposed residues can alter receptor binding and biological activity (14). The present homology model cannot determine whether A48 affects the interaction of TBIFN-β with IFNAR1 or IFNAR2. Direct comparison of wild-type TBIFN-β with residue-48 mutants, together with receptor-binding and downstream signaling assays, will be necessary to determine its functional significance.

Analysis of the TBIFNB upstream region identified predicted binding sites for IRFs and NF-κB. Both the −900–0 and −500–0 promoter fragments responded to batIRF1 expression and poly(I:C) treatment. The retained activity of the −500–0 construct suggests that the proximal 500-bp region contains regulatory information sufficient to support promoter activation under the tested conditions. This finding is consistent with evidence that IRF1 and IRF7 regulate antiviral gene expression in bat cells (7, 8). In other mammals, IFNB transcription depends on cooperative recruitment of IRFs, NF-κB, and additional transcription factors to the promoter enhanceosome (15). However, the sites identified in the present study remain computational predictions. Reporter activation does not demonstrate direct binding of batIRF1 to a specific site or verify the function of individual IRF and NF-κB motifs. Site-directed promoter mutagenesis, chromatin immunoprecipitation, or electrophoretic mobility-shift assays would be required for site-specific validation.

The poly(I:C) concentration and time-course experiments further supported the use of the cloned TBIFNB promoter for monitoring promoter activation. The TBIFNB reporter showed a modest but measurable response in Tb 1 Lu cells, with the highest observed activity at 6 h. Parallel experiments using huIFNB promoter reporters in HEK293T and HeLa cells produced larger observed induction amplitudes. These findings should not be interpreted as evidence that the human promoter is intrinsically more sensitive than the bat promoter. The assays used different cell lines, species-matched promoter constructs, and potentially different transfection efficiencies and poly(I:C)-sensing capacities. The TBIFNB construct should therefore be described as a functionally validated proof-of-concept reporter rather than a fully optimized analytical platform. A more complete evaluation would require broader concentration ranges, specificity controls, analysis of raw Firefly and Renilla signals, and formal assessment of intra-assay and inter-assay reproducibility.

Transfection with the TBIFN-β expression construct markedly reduced VSV-GFP and VACV-GFP fluorescence and decreased VSV-NP and VACV-C23L transcript copy equivalents. These results are consistent with previous evidence that IFN-β from *Myotis davidii* has antiviral activity and with recent observations of species-associated bat IFN-β responses (3, 16). However, the present findings should be described as antiviral activity in several tested RNA and DNA virus infection models rather than broad-spectrum antiviral activity.

Flag-tagged TBIFN-β was detected in both cellular lysates and concentrated conditioned-medium samples. GAPDH was detected in cellular lysates but not in conditioned medium, consistent with minimal detectable contamination by intracellular proteins. In addition, conditioned medium from TBIFN-β-transfected cells induced STAT1 phosphorylation in recipient cells, providing functional evidence that the conditioned medium contained extracellularly detectable material capable of activating interferon-associated signaling. Nevertheless, these experiments did not quantify the concentration, processing, secretion efficiency, receptor-binding affinity, or specific activity of TBIFN-β. Furthermore, GFP fluorescence and viral transcript copy equivalents do not directly measure infectious progeny production. Future studies should therefore include purified recombinant TBIFN-β, quantitative protein assays, receptor-binding analyses, and infectious-virus titration.

TBIFN-β induced distinct ISG profiles depending on the infection context. OAS1 was significantly increased under mock, VSV-GFP, VACV-GFP, and IAV-H1N1 conditions and showed the strongest response in the ISG promoter assays. This result is broadly consistent with observations in *Pteropus alecto*, in which OAS1 and Mx1 were highly inducible by interferon or viral infection, whereas PKR showed a different regulatory pattern (17, 18). However, the endogenous-gene and promoter-reporter results in the present study were not fully concordant. PKR mRNA increased under some conditions even though the cloned PKR promoter was not significantly activated. Conversely, the Mx1 promoter showed modest activation even when endogenous Mx1 expression was unchanged in some infection groups. These differences may reflect distal regulatory elements, chromatin context, indirect transcriptional regulation, or changes in mRNA stability. They may also indicate that the cloned promoter fragments did not contain all regulatory sequences controlling endogenous gene expression.

Consistent with this interpretation, RNA sequencing identified a broader transcriptional program involving viral sensors, signaling regulators, and antiviral effector genes. The RNA-seq OAS transcript could not be confidently assigned to a specific subtype and should not be assumed to represent the same OAS1 transcript measured using targeted RT-qPCR.

Pyridone 6 treatment substantially attenuated TBIFN-β-associated induction of STAT1 and several ISGs and partially reversed the suppression of VSV-GFP and VACV-GFP. The conditioned-medium experiment provided additional protein-level support, as TBIFN-β-Flag-conditioned medium induced STAT1 phosphorylation at 30 and 60 min, whereas this response was markedly attenuated following Pyridone 6 pretreatment. Together, these findings support an important contribution of JAK-dependent signaling to the effects associated with the TBIFN-β expression construct.

Pyridone 6 is a pan-JAK inhibitor and cannot distinguish the contributions of individual JAK family members (19). The immunoblotting results were also evaluated qualitatively without densitometric quantification. Therefore, the data support Pyridone 6-sensitive STAT1 phosphorylation but do not establish JAK1-specific signaling or quantify the extent of pathway inhibition. Moreover, the persistence of antiviral activity after Pyridone 6 treatment does not demonstrate a JAK-independent TBIFN-β pathway. Residual activity could result from incomplete pathway inhibition, insufficient inhibitor exposure, or secondary cellular responses. Further studies using validated *T. brasiliensis*-specific genetic perturbation, STAT2 phosphorylation analysis, receptor-blocking experiments, and rescue assays will be required to define the contributions of individual JAK and STAT proteins.

RNA sequencing provided a broader view of the transcriptional response associated with TBIFN-β expression. GO enrichment highlighted immune response, cytokine-responsive processes, innate immune response, interferon-mediated signaling, and type I interferon-associated responses. KEGG analysis of the upregulated gene set further identified JAK-STAT signaling, RIG-I-like receptor signaling, Toll-like receptor signaling, cytosolic DNA-sensing, NF-κB signaling, and TNF signaling among the displayed enriched pathways. These pathway-level findings were consistent with the upregulation of viral sensors, including RIG-I, MDA5, LGP2, and TLR3; signaling regulators, including IRF7, IRF9, STAT1, STAT2, and TRIM25; and antiviral effectors, including an OAS-family transcript, ISG15, IFIT3, Mx1, GBP2, and RNaseL.

RT-qPCR confirmed the direction of change for IRF7, MDA5, ISG15, and STAT1. Together with the targeted ISG experiments, these findings support activation of a coordinated interferon-associated antiviral program rather than isolated regulation of a small number of downstream genes. However, enrichment analysis identifies the overrepresentation of genes within annotated categories and does not demonstrate direct activation of every component of the corresponding pathways.

IFNW, RANK, and cIAP1/2 were among the selected downregulated immune-related transcripts. KEGG analysis of the downregulated gene set also identified cytokine-cytokine receptor interaction, C-type lectin receptor signaling, chemokine signaling, IL-17 signaling, and complement and coagulation cascades among the displayed pathway categories. The reduction in IFNW is notable because unconventional IFNω-like genes represent an important component of type I interferon loci in several bat species (9). This change may reflect cross-regulation among type I interferon subtypes, but the current data do not demonstrate a negative-feedback mechanism. Similarly, enrichment of pathway categories among downregulated genes and reduced RANK or cIAP1/2 expression cannot by themselves establish global immune suppression, regulation of apoptosis, or protection from immunopathology (20). Functional perturbation and protein-level analyses would be required before such mechanisms could be assigned to TBIFN-β.

Several limitations should be considered. First, all functional characterization of TBIFN-β in this study, including antiviral assays, downstream ISG analyses, pharmacological inhibition, conditioned-medium signaling experiments, and transcriptomic profiling, was performed using the single immortalized Tb 1 Lu cell line. Although this cell line provides a useful *in vitro* system for studying *T. brasiliensis* antiviral responses, immortalization and tissue origin may influence cellular signaling and gene-expression patterns. Therefore, the present findings cannot be assumed to represent responses in primary *T. brasiliensis* cells, other bat tissues, or the *in vivo* physiological context. Validation in primary bat cells and, where feasible, *in vivo* models will be necessary to establish the physiological relevance and generalizability of these findings. Second, much of the functional characterization relied on plasmid-mediated overexpression of TBIFN-β. Although this approach allowed controlled evaluation of antiviral activity and downstream signaling, the level and duration of TBIFN-β expression may not fully reflect endogenous IFN-β production or physiological cytokine exposure. Future studies should therefore validate these findings using primary *T. brasiliensis* cells, quantitatively defined recombinant TBIFN-β, endogenous genetic perturbation approaches, additional bat species, and *in vivo* models where feasible. Third, TBIFN-β protein expression and extracellular detection were assessed qualitatively through a C-terminal Flag tag. The concentration, native processing, secretion efficiency, receptor-binding affinity, and specific biological activity of TBIFN-β were not quantified. Fourth, the functional relevance of residue 48, the identity of individual promoter response elements, and direct receptor binding were not experimentally determined. Fifth, the reporter system underwent functional testing but not complete analytical validation for sensitivity, specificity, dynamic range, or inter-assay reproducibility. Sixth, viral infection was assessed mainly through GFP fluorescence and viral transcript measurements rather than infectious-virus yield. Seventh, the STAT1 phosphorylation assay supported pathway activation and Pyridone 6 sensitivity but did not distinguish individual JAK family members, evaluate STAT2 phosphorylation, or provide quantitative analysis of phosphorylation. To further examine the role of STAT1, we also performed preliminary siRNA-mediated STAT1 interference experiments and evaluated STAT1 expression by RT-qPCR and immunoblotting. Several candidate siRNAs produced partial reductions in STAT1 expression; however, the magnitude of the interference varied among the siRNAs and between the plasmid co-transfection and conditioned-medium treatment conditions. Moreover, the downstream OAS1 response did not show a consistent reduction following STAT1 interference. We therefore considered these results preliminary and did not use them as formal evidence of STAT1 dependence in the present study. One possible limitation is the relatively limited availability and annotation of species-specific genomic and transcript sequence information for *T. brasiliensis*, together with the lack of validated gene-silencing reagents for Tb 1 Lu cells. Future work will include further verification of the STAT1 target sequence, screening and optimization of additional species-specific siRNAs, optimization of transfection and sampling conditions, and complementary genetic approaches such as genome editing and rescue experiments where feasible. Eighth, the transcriptomic analysis was based on *de novo* assembly and incomplete gene annotation, and the biological implications of the enriched pathways require additional functional validation. Finally, reliable protein-level analysis of Mx1 and PKR could not be performed because the commercially available antibodies tested did not produce specific bands at the expected molecular weights in Tb 1 Lu cells, possibly because of sequence divergence between the *T. brasiliensis* proteins and the antigens used to generate these antibodies. Future studies will require the generation and validation of *T. brasiliensis*-specific antibodies to evaluate the protein-level regulation of these antiviral effectors.

In conclusion, this study provides an integrated *in vitro* characterization of TBIFN-β and establishes experimental tools for examining its promoter activity and downstream responses. TBIFN-β retained a conserved predicted cytokine fold, was detected in conditioned medium, induced Pyridone 6-sensitive STAT1 phosphorylation in recipient cells, activated an interferon-associated transcriptional network, and was associated with reduced infection in several viral models in Tb 1 Lu cells. OAS1 was the most consistently induced of the three targeted ISGs, whereas PKR and Mx1 showed more context-dependent regulation. GO and KEGG enrichment analyses further connected the transcriptional response with cytokine signaling, viral recognition, and antiviral effector pathways. Validation using purified protein, primary cells, additional bat species, and *in vivo* systems will be required before these findings can be extended to physiological infection or viral tolerance at the whole-animal level.

## Data Availability

The RNA-sequencing data generated in this study have been deposited in the NCBI Sequence Read Archive (SRA) under BioProject accession PRJNA1517831 and SRA Study accession SRP730344.
