# Supplementary material for: In vitro functional characterization of Tadarida brasiliensis interferon-β: promoter responsiveness, antiviral activity, and JAK-dependent transcriptional responses
Source: Front Immunol. 2026 Sep 3;17:1915149. doi: 10.3389/fimmu.2026.1915149 (PMC13581577; doi:10.3389/fimmu.2026.1915149)
Supplement: Supplementary file 1 [file DataSheet1.pdf]

Supplementary Material

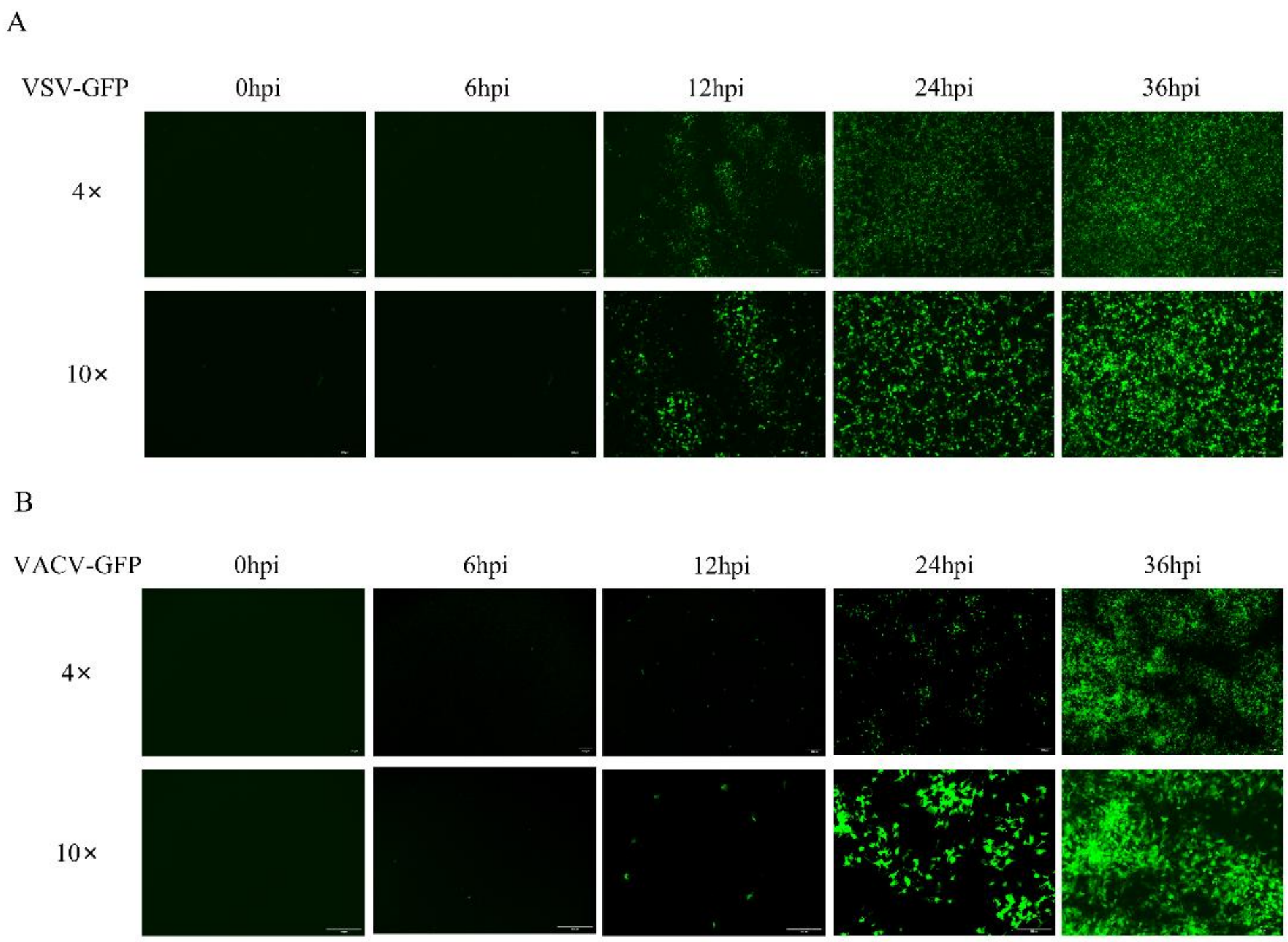

Supplementary Figure S1. Time-dependent progression of VSV-GFP and VACV-GFP infection in Tb 1 Lu cells. Tb 1 Lu cells were infected with VSV-GFP at an MOI of 0.001 or VACV-GFP at an MOI of 0.1. GFP fluorescence was monitored at 0, 6, 12, 24, and 36 h post-infection (hpi). (A) Representative fluorescence microscopy images of VSV-GFP-infected cells acquired using 4× and 10× objectives. (B) Representative fluorescence microscopy images of VACV-GFP-infected cells acquired using 4× and 10× objectives. Scale bars are shown in the individual images. Images acquired at the same magnification were collected using fixed GFP-channel acquisition settings across the corresponding experimental groups. GFP fluorescence was used for qualitative visualization and was not subjected to quantitative fluorescence-intensity analysis.

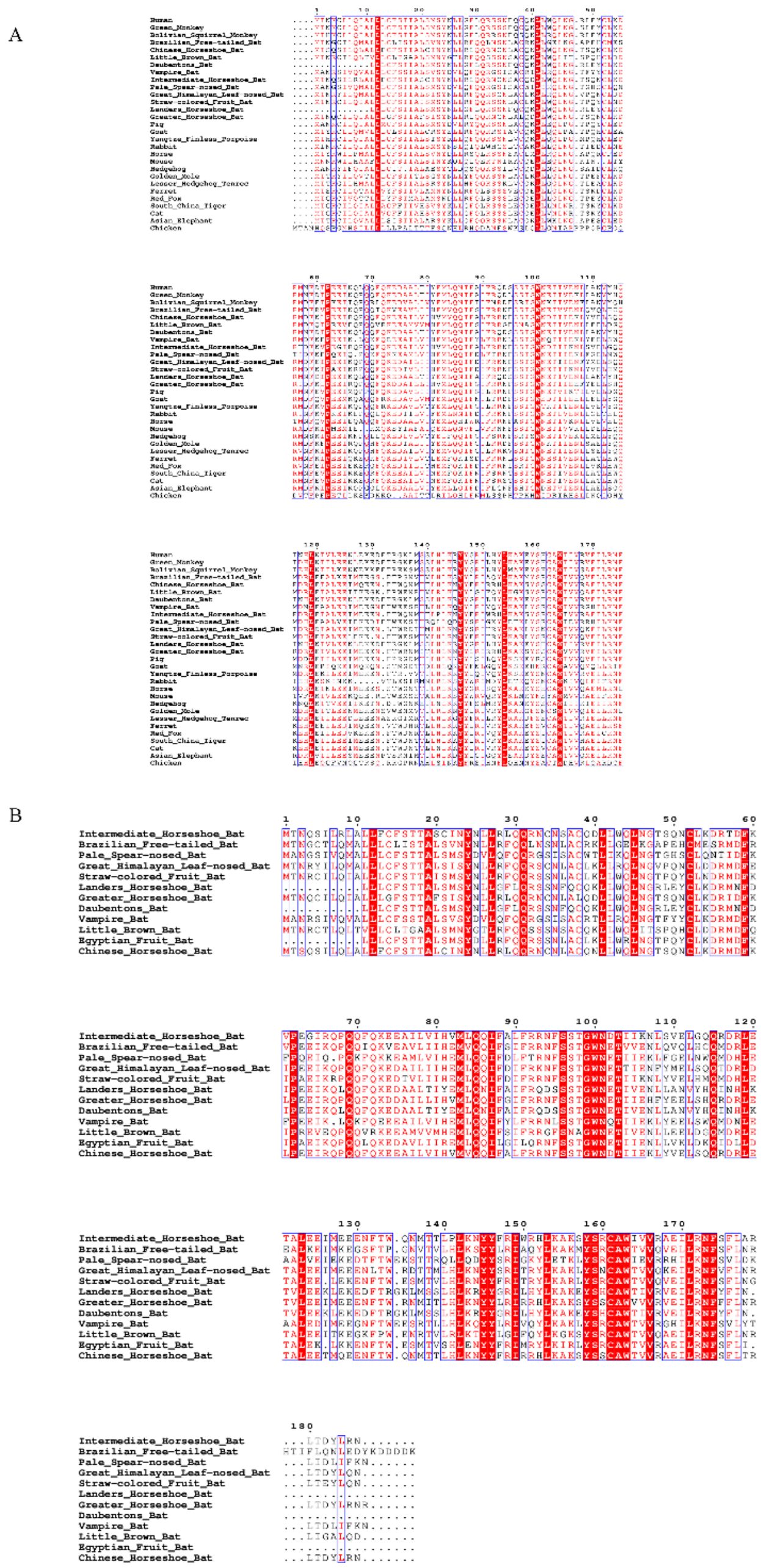

Supplementary Figure S2. Full-length multiple sequence alignments of mammalian and bat IFN-β proteins. (A) Full-length amino acid sequence alignment of IFN-β proteins from representative mammalian species, with chicken included as an outgroup. (B) Full-length amino acid sequence alignment of IFN-β proteins from representative bat species. Amino acid positions are numbered according to the full-length precursor proteins. The highlighted column indicates residue 48. In panel A, alanine at residue 48 was present in *Tadarida brasiliensis*, horse, and Asian elephant. Among the bat sequences shown in panel B, *T. brasiliensis* contained alanine at this position. Residues are colored according to sequence conservation, and dots indicate alignment gaps introduced to optimize sequence comparison.

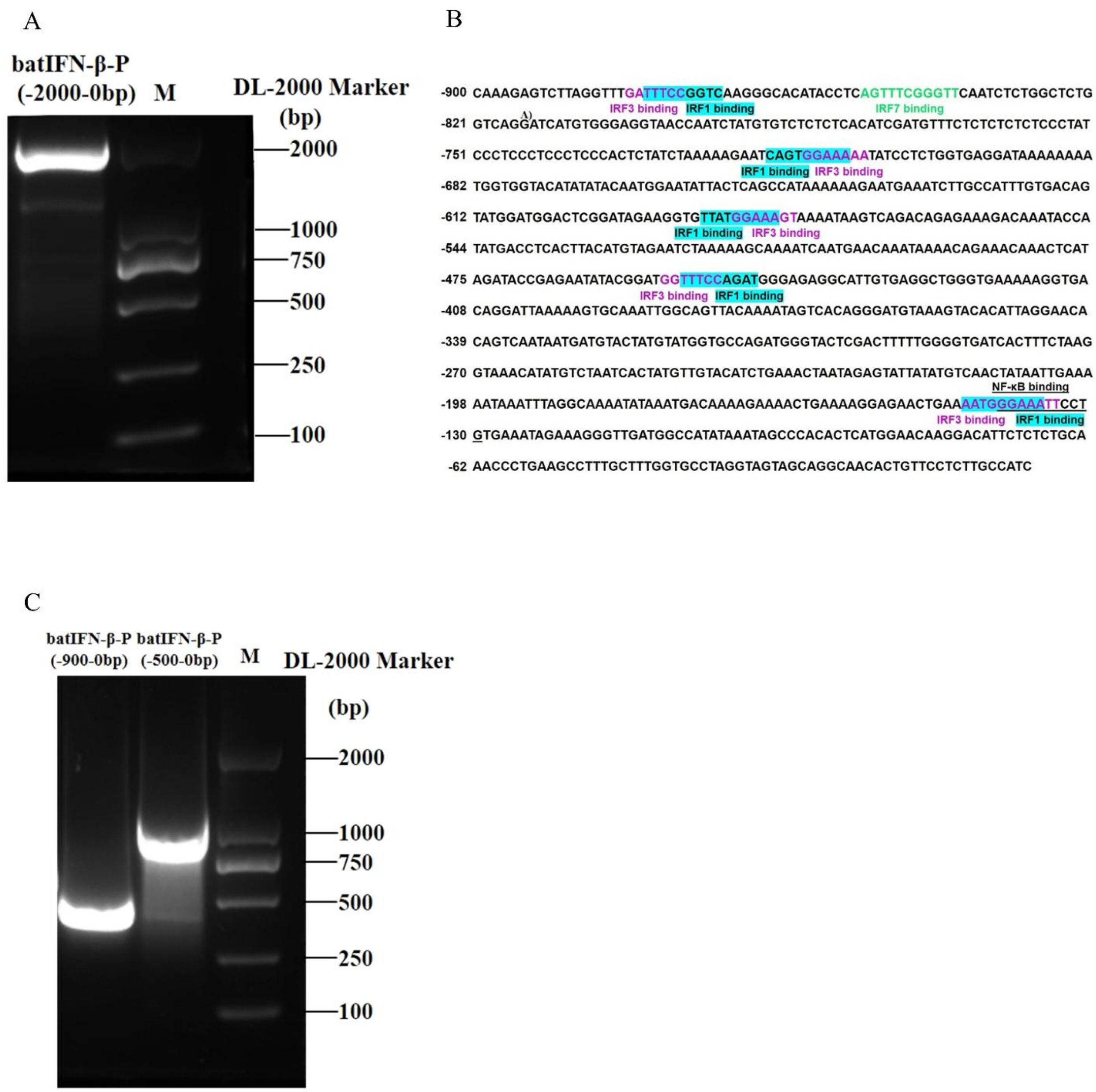

Supplementary Figure S3. Amplification and sequence analysis of the TBIFNB promoter fragments used for reporter construction.
(A) Agarose gel electrophoresis of the approximately 2,000-bp genomic region upstream of the TBIFNB translation start site amplified from Tb 1 Lu cell genomic DNA.
(B) Nucleotide sequence of the −900/0 TBIFNB promoter region used for reporter construction. Predicted binding sites for IRF1, IRF3, and NF-κB are highlighted and labeled. Nucleotide positions are shown relative to the translation start site, with the ATG codon defined as position 0.
(C) Agarose gel electrophoresis of the amplified −900/0 and −500/0 TBIFNB promoter fragments. M, DL2000 DNA marker; bp, base pairs.

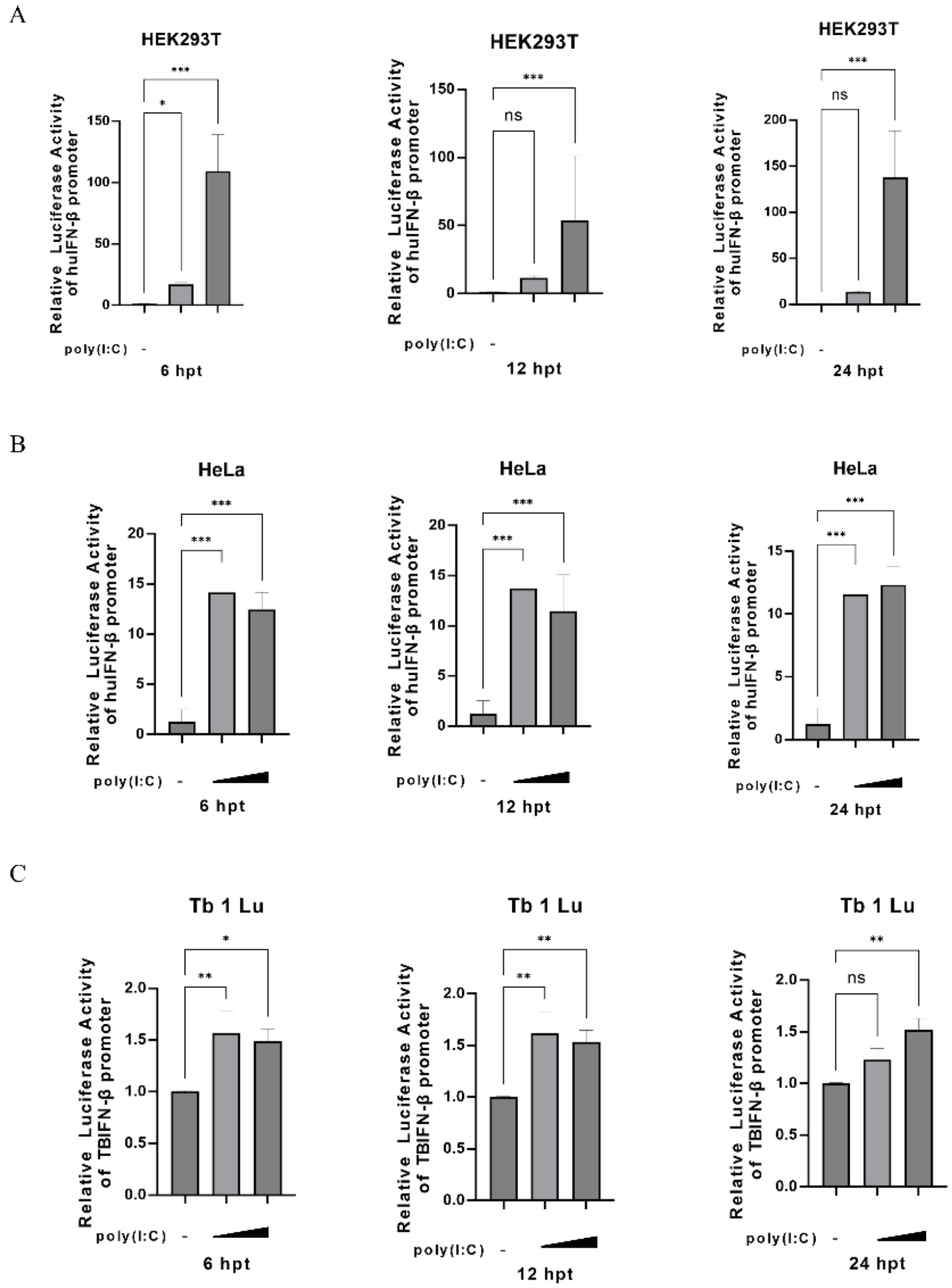

Supplementary Figure S4. Time- and concentration-dependent activation of human and bat IFNB promoter reporters by poly(I:C).

HEK293T, HeLa, and Tb 1 Lu cells were transfected with the corresponding IFNB promoter firefly luciferase reporter construct together with the pRL-TK Renilla luciferase internal-control plasmid. Cells were subsequently treated with 0.5 or 1 μg/mL poly(I:C), and dual-luciferase activity was measured at 6, 12, and 24 h after treatment.

(A) Time- and concentration-dependent activation of the huIFNB promoter reporter in HEK293T cells.

(B) Time- and concentration-dependent activation of the huIFNB promoter reporter in HeLa cells.

(C) Time- and concentration-dependent activation of the TBIFNB promoter reporter in Tb 1 Lu cells.

Firefly luciferase activity was normalized to Renilla luciferase activity, and the relative reporter activity was calculated using the corresponding untreated control as the calibrator. Data are presented as the mean ± SD from three independent experiments (n = 3), each performed with three technical replicate wells. Technical replicates were averaged within each independent experiment before statistical analysis, and error bars represent SD. Statistical significance was assessed using one-way

ANOVA followed by Dunnett's multiple-comparisons test, with each poly(I:C)-treated group compared with the corresponding untreated control at the same time point. ns, not significant; *$P < 0.05$; **$P < 0.01$; ***$P < 0.001$. Because different cell lines and species-matched promoter constructs were used, the results are presented as descriptive benchmarking and were not used for direct statistical comparison of intrinsic promoter sensitivity between the human and bat reporter systems.

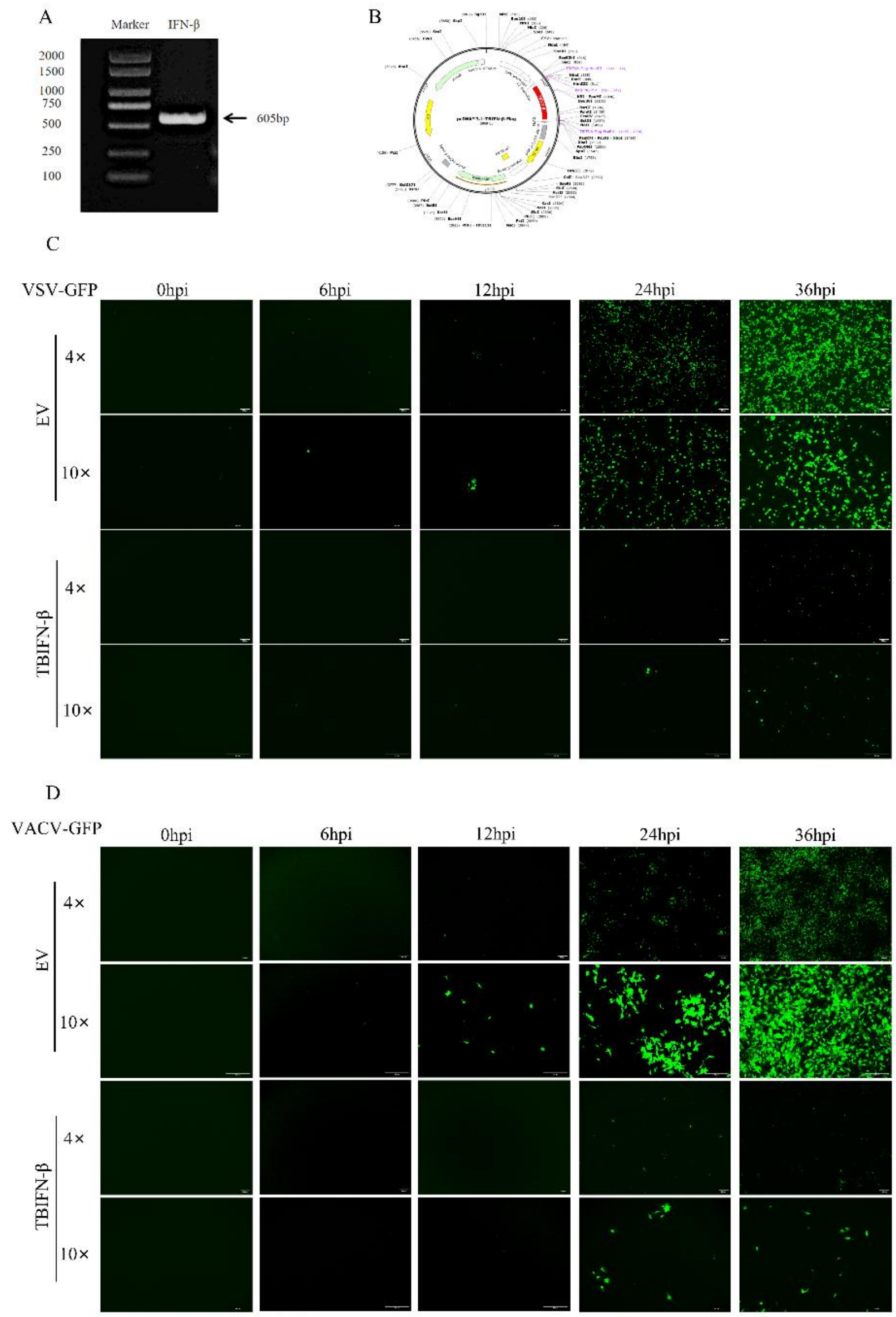

Supplementary Figure S5. Construction of the TBIFN-β expression plasmid and fluorescence evaluation of its antiviral activity against VSV-GFP and VACV-GFP.

(A) Agarose gel electrophoresis of the amplified TBIFNB coding sequence. The PCR product of the expected size was used for construction of the expression plasmid. M, DNA marker.

(B) Schematic map of the pcDNA3.1-TBIFN-β-Flag expression plasmid, in which the TBIFNB coding sequence was inserted in frame with a C-terminal Flag tag.

(C) Complete fluorescence microscopy time course of VSV-GFP infection in Tb 1 Lu cells transfected with either the empty vector (EV) or pcDNA3.1-TBIFN-β-Flag. Images were acquired at 0, 6, 12, 24, and 36 h post-infection (hpi) using 4× and 10× objectives.

(D) Complete fluorescence microscopy time course of VACV-GFP infection in Tb 1 Lu cells transfected with EV or pcDNA3.1-TBIFN-β-Flag. Images were acquired at 0, 6, 12, 24, and 36 hpi using 4× and 10× objectives. Scale bars are shown in the individual images. Images acquired at the

same magnification were collected using fixed GFP-channel acquisition settings across the corresponding experimental groups. GFP fluorescence was used for qualitative visualization and was not subjected to quantitative fluorescence-intensity analysis.

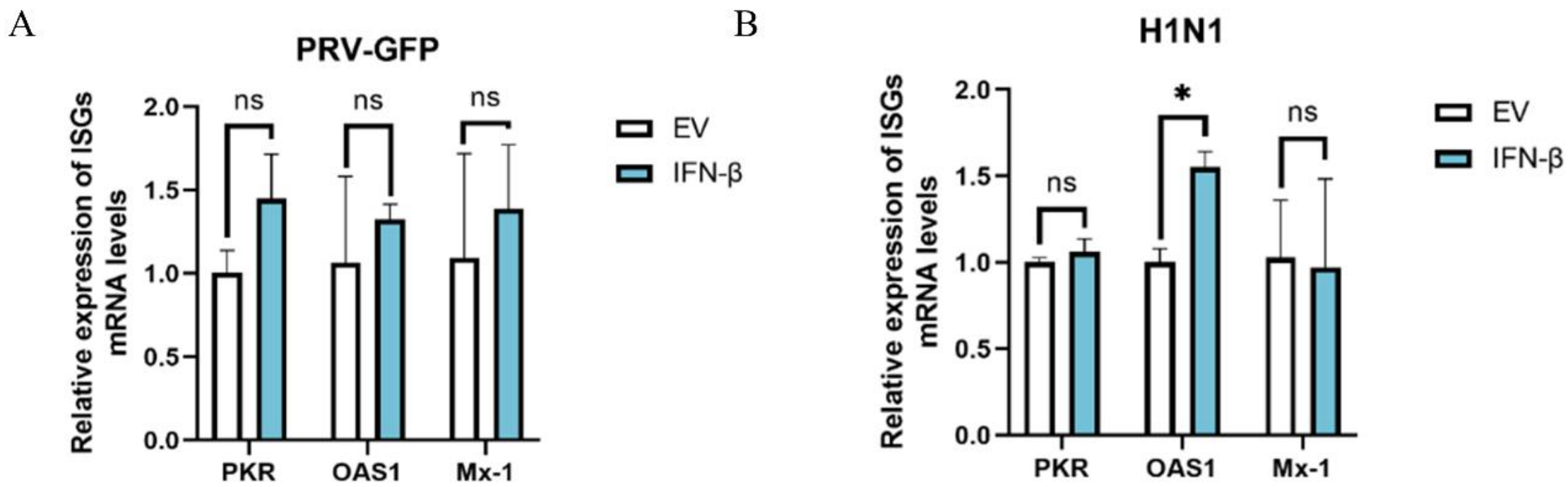

Supplementary Figure S6. ISG expression following PRV-GFP or IAV-H1N1 infection in cells transfected with the TBIFN-β expression construct.

Tb 1 Lu cells were transfected with either the empty vector (EV) or pcDNA3.1-TBIFN-β-Flag for 24 h and were subsequently infected with PRV-GFP or IAV-H1N1. Samples were collected at 24 h post-infection for RT-qPCR analysis.

(A) Relative mRNA levels of PKR, OAS1, and Mx1 in EV- and pcDNA3.1-TBIFN-β-Flag-transfected cells following PRV-GFP infection.

(B) Relative mRNA levels of PKR, OAS1, and Mx1 in EV- and pcDNA3.1-TBIFN-β-Flag-transfected cells following IAV-H1N1 infection. Transcript levels were normalized to β-actin and calculated using the 2^−ΔΔCt method, with the corresponding EV group used as the calibrator. Data are presented as the mean ± SD from three independent experiments (n = 3), and error bars represent SD. Panels A and B were analyzed using two-way ANOVA followed by Sidak's multiple-comparisons test; *P < 0.05; **P < 0.01; ***P < 0.001.

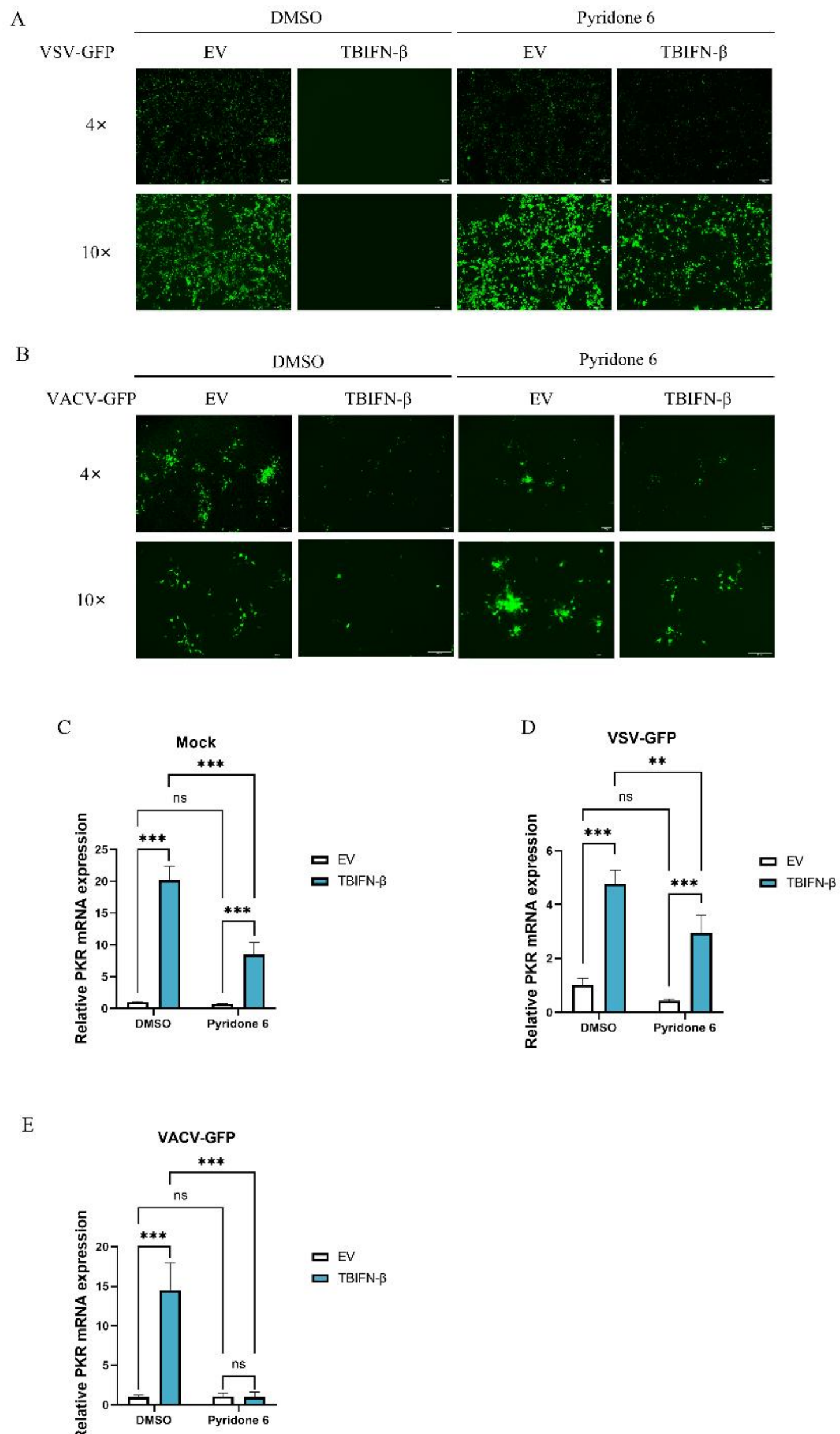

Supplementary Figure S7. Effects of Pyridone 6 on TBIFN-β-associated antiviral activity and PKR expression. Tb 1 Lu cells were transfected with either the empty vector (EV) or pcDNA3.1-TBIFN-β-Flag. At 24 h after transfection, cells were pretreated with 10 μM Pyridone 6 or the corresponding DMSO vehicle for 2 h and were subsequently mock infected or infected with VSV-GFP at an MOI of 0.001 or VACV-GFP at an MOI of 0.1. Pyridone 6 or DMSO remained present throughout the infection period.
(A) Representative fluorescence microscopy images of VSV-GFP-infected cells at 24 h post-infection. Images were acquired using 4× and 10× objectives.
(B) Representative fluorescence microscopy images of VACV-GFP-infected cells at 24 h post-infection. Images were acquired using 4× and 10× objectives. Scale bars are shown in the individual images.
(C–E) Relative PKR mRNA levels under mock conditions (C), following VSV-GFP infection (D),

and following VACV-GFP infection (E). Samples were collected at 24 h post-infection. PKR transcript levels were normalized to β-actin and calculated using the $2^{-\Delta\Delta Ct}$ method. Within each condition, the EV plus DMSO group was used as the calibrator. Data are presented as the mean ± SD from three independent experiments (n = 3), and error bars represent SD. Statistical significance was assessed using two-way ANOVA followed by Sidak's multiple-comparisons test. ns, not significant; *$P < 0.05$; **$P < 0.01$; ***$P < 0.001$.

A

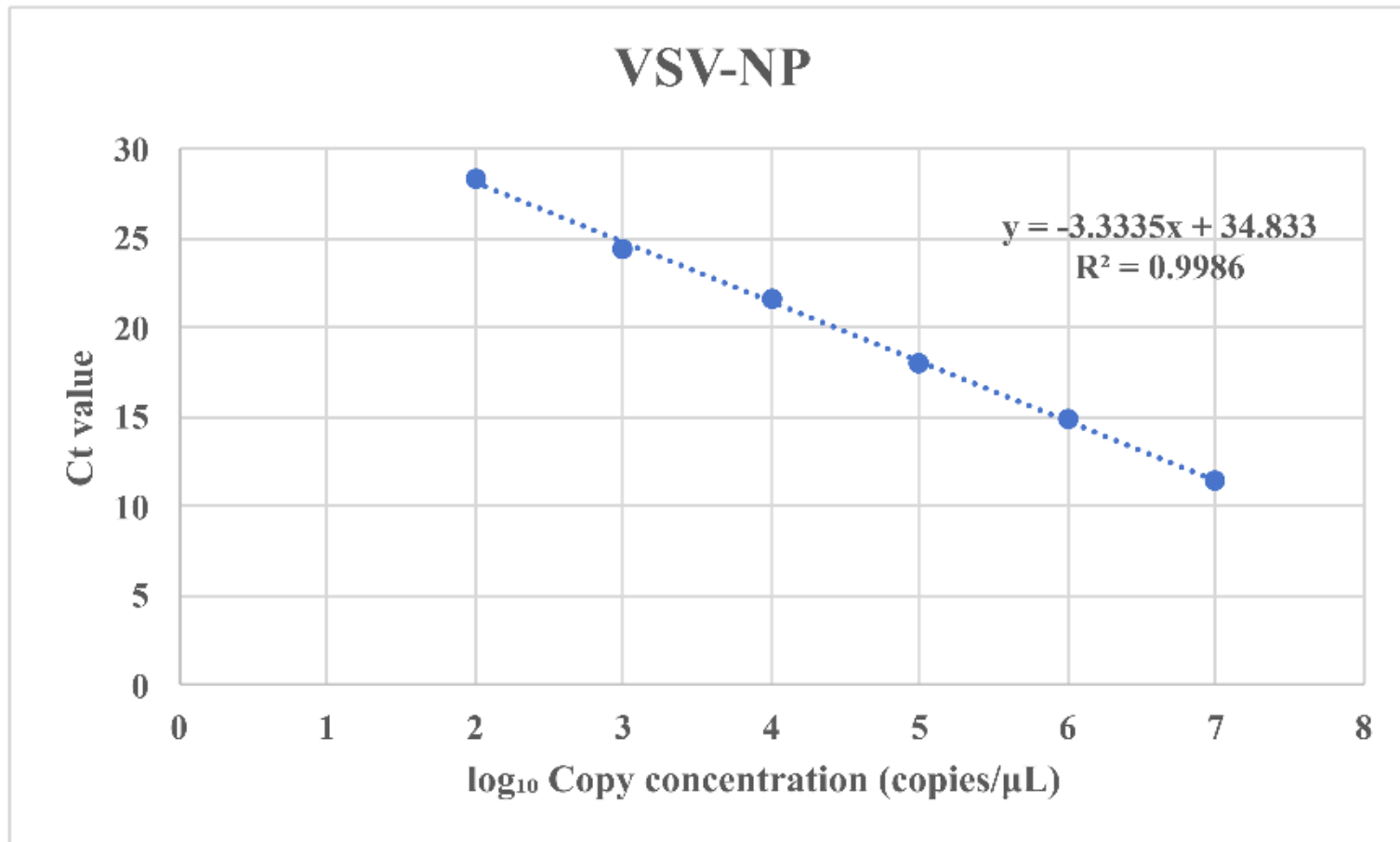

B

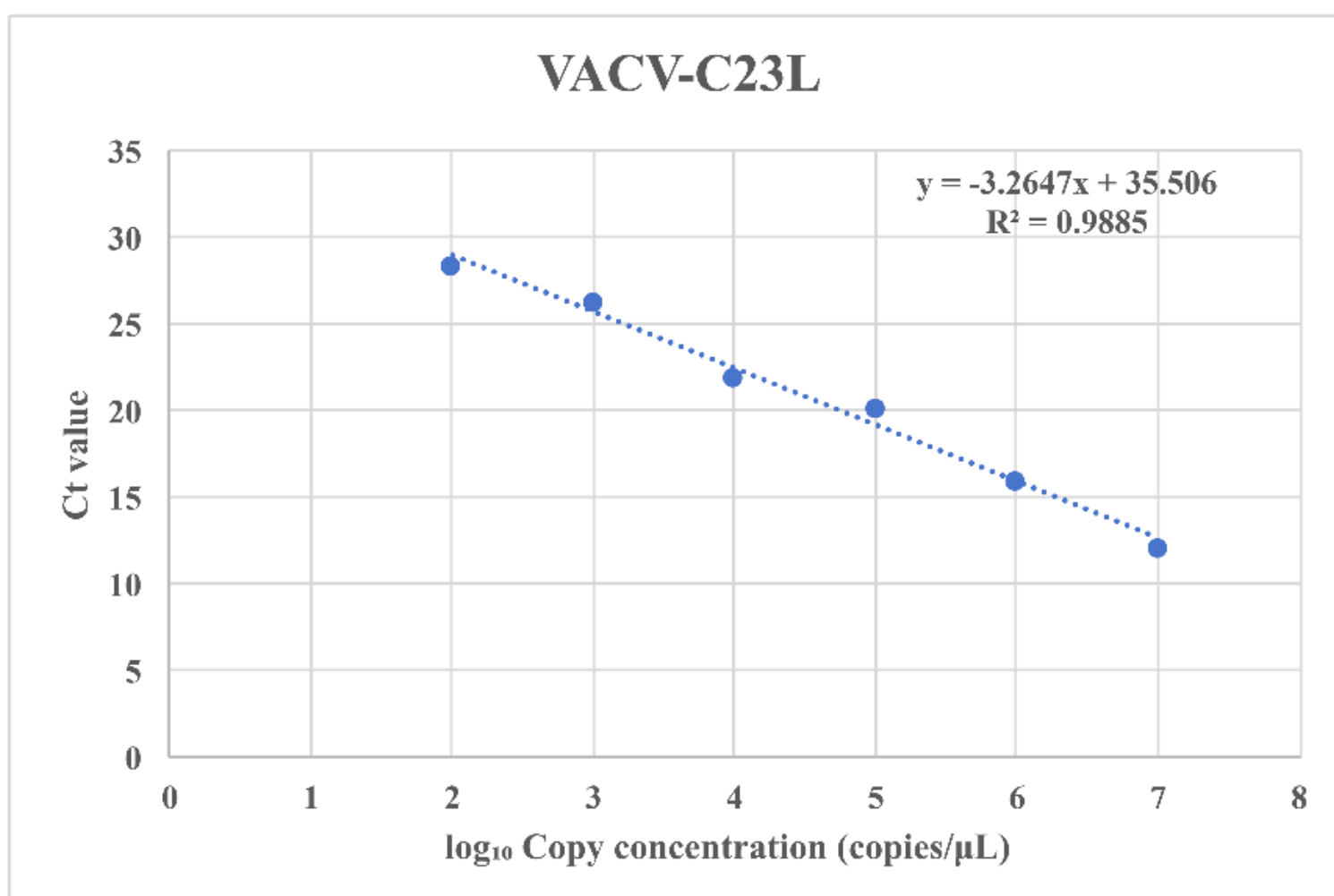

Supplementary Figure S8. External standard curves for absolute quantification of VSV-NP and VACV-C23L transcript copy equivalents. Purified PCR products corresponding to a 183-bp fragment of VSV-NP and a 146-bp fragment of VACV-C23L were used as external standards. Serial dilutions ranging from 10^2 to 10^7 copies/μL were analyzed by quantitative real-time PCR under the same amplification conditions used for the experimental samples. Standard curves were generated by linear regression of the Ct values against the $\log_{10}$-transformed standard copy concentrations.

(A) External standard curve for VSV-NP. The regression equation was $Ct = -3.3335 \log_{10}(C) + 34.8332$, with an R^2 value of 0.9986 and an amplification efficiency of 99.5%.

(B) External standard curve for VACV-C23L. The regression equation was $Ct = -3.2647 \log_{10}(C) + 35.5060$, with an R^2 value of 0.9885 and an amplification efficiency of 102.4%.

C represents the standard copy concentration in copies per microliter. The standard curves were used to convert Ct values obtained from experimental samples into viral transcript copy equivalents.

Supplementary Table S1. Primers used in this study

Genes	Primer	Nucleotide sequence of primers (5'-3')
TBIFN-β	Forward	ATGACCAATGGATGCACTCTCCAGAT
	Reverse	TCAGTTTTGGAGGAAAATCG
pcDNA3.1-TBIFN-β	Forward	CTGGCTAGCGTTTAAACTTAAGCTTATGACCAATGGATGCAC
	Reverse	GGCCGCCACTGTGCTGGATATCGTTTTGGAGGAAAATCGTAT
TBIFN-β-Promoter (-2000 to 0 bp)	Forward	ACTGAATCAAGTTACTCCACC
	Reverse	GATGGCAAGAGGAACAGTGTT
pGL3.0-basic (reverse PCR)	Forward	AGATCTGCGATCTAAGTAAGCTTGG
	Reverse	GCTAGCACGCGTAAGAGCTC
pGL3.0-TBIFN-β-(-900 to 0 bp)	Forward	CGAGCTCTTACGCGTGCTAGCCCCAGTAATTATACTTCTGCT
	Reverse	GCTTACTTAGATCGCAGATCTGATGGCAAGAGGAACAGTGTT
pGL3.0-TBIFN-β-(-500 to 0 bp)	Forward	CGAGCTCTTACGCGTGCTAGCAACAAATAAAACAGAAACAAA
	Reverse	GCTTACTTAGATCGCAGATCTGATGGCAAGAGGAACAGTGTT
TBMx-1-Promoter (-2000 to 0 bp)	Forward	GTAGATAAGAAGGCAAAGCCTGGCCCTT
	Reverse	GATGTGCCCTGTCTGCAGCT
TBOAS1-Promoter (-2000 to 0 bp)	Forward	GGGCGCAACTCAGCTCCAGACAAATTT
	Reverse	CGAAACTTACTCTCTGTCTTTAACATCAGGC
TBPKR-Promoter (-2000 to 0 bp)	Forward	AATTCCTCACTTAGGCAAGAGATC
	Reverse	ACCCCCTGAGTGACAGCCTCCCTCGGACAT

pGL3.0-TBMx-1	Forward	CGAGCTCTTACGCGTGCTAGCGTAGATAAGAAGGCAAAGCCT
	Reverse	GCTTACTTAGATCGCAGATCTGATGTGCCCTGTCTGCAGCTG
pGL3.0-TBOAS1	Forward	CGAGCTCTTACGCGTGCTAGCGGGCGCAACTCAGCTCCAGAC
	Reverse	GCTTACTTAGATCGCAGATCTCGAAACTTACTCTCTGTCTTT
pGL3.0-TBPKR	Forward	CGAGCTCTTACGCGTGCTAGCAATTCCTCACTTAGGCAAGAG
	Reverse	GCTTACTTAGATCGCAGATCTACCCCCTGAGTGACAGCCTCC
qTBMx-1	Forward	GGAGGGTCAGCTCCCCTCA
	Reverse	GCCATGCTCAGCGCCTCT
qTBPKR	Forward	GGATTGCCCAAAAGGCAA
	Reverse	GGTAGGACCTGTAGCATCA
qTBIFN-β	Forward	TGCACTCTCCAGATGGCT
	Reverse	CCTGCTCTCCATGCAATGT
qTBOAS1	Forward	ATCTGCAGTTTCCTGAAGGAG
	Reverse	GCTGAGGAAGCGACGAGGTC
qTBβ-actin	Forward	CCATCCTGCGTCTGGACCTGG
	Reverse	GTGGCCATCTCCTGCTCGAAG
qVSV-NP	Forward	AGTGGAATACCCGGCAGATTAC
	Reverse	TCCAACTTACCCCGGATGTC
qVACV-C23L	Forward	TCAGACACACGCTTTGAG
	Reverse	TTAGCGTTCGTATCGGAG

qPRV-UL54	Forward	GTACTTCCCGTGCATGTACACG
	Reverse	TCCCGCTGGAGGAGTACGTG
qH1N1-NP	Forward	CAGATACTGGGCCATAAGGAC
	Reverse	GCATTGTCTCCGAAGAAATAAG
qTBSTAT1	Forward	CAGACCCCAGACCTAGCAGA
	Reverse	CAGCTGCGATAGGAGGTCAT
qTBRANK	Forward	GCCTTGCCTCGTAGGCTATT
	Reverse	CCCAAGACTGGTACAGTTGGT
qTBISG15	Forward	CTGAAACTCAAATCCCCGCC
	Reverse	TCACGTACCCGTTCATGGCT
qTBIRF7	Forward	TTCCGCTGCGCCCTGAGCAGC
	Reverse	CTTCAAGGGTCTTGCTGT
qTBIFN-ω	Forward	ACCAACTGTGCAGGGCA
	Reverse	CTCTTCAAGGCCAGTGTAG
qTBMDA5	Forward	TTCAGCCCACTCTGGTGGACA
	Reverse	TCTCTTTCTGCACAATCCTTC

Supplementary Table S2. IFN-β protein sequences used for phylogenetic and multiple sequence analyses.

No.	Taxonomic order	Scientific name	Database	Identifier reported in original manuscript	Identifier type
1	Primates	*Homo sapiens*	NCBI Gene	3456	Gene ID
2	Primates	*Chlorocebus sabaeus*	NCBI Gene	103219383	Gene ID
3	Primates	*Saimiri boliviensis*	NCBI RefSeq	XP_003928175.3	Protein accession
4	Chiroptera	*Tadarida brasiliensis*	NCBI Gene / sequence obtained in this study	PZ798294	GenBank accession
5	Chiroptera	*Desmodus rotundus*	UniProtKB	A0A2H4P4H5	Protein accession
6	Chiroptera	*Rousettus aegyptiacus*	NCBI Gene	107518589	Gene ID
7	Cetartiodactyla	*Capra hircus*	NCBI Gene	102181595	Gene ID
8	Cetartiodactyla	*Neophocaena asiaeorientalis asiaeorientalis*	NCBI Gene	116724119	Gene ID
9	Cetartiodactyla	*Sus scrofa*	NCBI Gene	445459	Gene ID
10	Perissodactyla	*Equus caballus*	NCBI Gene	100052545	Gene ID
11	Lagomorpha	*Oryctolagus cuniculus*	NCBI Gene	100342931	Gene ID
12	Rodentia	*Mus musculus*	NCBI Gene	15977	Gene ID
13	Carnivora	*Vulpes vulpes*	NCBI Gene	112921940	Gene ID
14	Carnivora	*Mustela putorius furo*	NCBI Gene	101679100	Gene ID
15	Carnivora	*Panthera tigris amoyensis*	NCBI Gene	102960421	Gene ID
16	Carnivora	*Felis catus*	NCBI Gene	493849	Gene ID
17	Proboscidea	*Elephas maximus indicus*	NCBI Gene	126083236	Gene ID
18	Afrosoricida	*Chrysochloris asiatica*	NCBI Gene	102820525	Gene ID

19	Afrosoricida	*Echinops telfairi*	NCBI Gene	101650550	Gene ID
20	Eulipotyphla	*Erinaceus europaeus*	NCBI Gene	103125201	Gene ID
21	Chiroptera	*Rhinolophus affinis*	GenBank	AJ25031.1	GenBank accession
22	Chiroptera	*Rhinolophus sinicus*	NCBI RefSeq	XP_019605912.2	Protein accession
23	Chiroptera	*Rhinolophus ferrumequinum*	NCBI Gene	117032413	Gene ID
24	Chiroptera	*Hipposideros armiger*	NCBI Gene	109396418	Gene ID
25	Chiroptera	*Eidolon helvum*	UniProtKB	A0A7S5W4K0	Protein accession
26	Chiroptera	*Rhinolophus landeri*	UniProtKB	H6U679	Protein accession
27	Chiroptera	*Myotis daubentonii*	NCBI Gene	132212752	Gene ID
28	Chiroptera	*Myotis lucifugus*	NCBI Gene	102417209	Gene ID
29	Chiroptera	*Phyllostomus discolor*	NCBI Gene	114542515	Gene ID
30	Galliformes	*Gallus gallus*	UniProtKB	Q90873	Protein accession

Supplementary Table S3. P values for statistical comparisons shown in the main and supplementary figures.

Figure	Panel	Comparison	Statistical test	P value
Figure 3	C	BatIRF1+pGL3.0-Basic vs BatIRF1+pGL-BatIFNB-P(-900bp)-luc	one-way ANOVA followed by Dunnett's multiple-comparisons test	2.39E-06
		BatIRF1+pGL3.0-Basic vs BatIRF1+pGL-BatIFNB-P(-500bp)-luc		2.74E-06
		poly(I:C)+pGL3.0-Basic vs poly(I:C)+pGL-BatIFNB-P(-900bp)-luc	one-way ANOVA followed by Dunnett's multiple-comparisons test	1.43E-07
		poly(I:C)+pGL3.0-Basic vs poly(I:C)+pGL-BatIFNB-P(-500bp)-luc		9.89E-07
	D	0 vs 0.5μg/mL	one-way ANOVA followed by Dunnett's multiple-comparisons test	0.0070
		0 vs 1μg/mL		0.0146
	E	0h vs 6h	one-way ANOVA followed by Dunnett's multiple-comparisons test	0.0001
		0h vs 12h		0.0008
		0h vs 24h		0.0011
Figure 4	B	EV vs TBIFN-β	two-tailed unpaired Student's t-test	<0.0001
	E	VSV-GFP-6h: EV vs TBIFN-β	two-way ANOVA followed by Sidak's multiple-comparisons test	<0.001
		VSV-GFP-12h: EV vs TBIFN-β		<0.001
		VSV-GFP-24h: EV vs TBIFN-β		<0.001
		VSV-GFP-36h: EV vs TBIFN-β		<0.001
	F	VACV-GFP-6h: EV vs TBIFN-β	two-way ANOVA followed by Sidak's multiple-comparisons test	>0.99
		VACV-GFP-12h: EV vs TBIFN-β		<0.001
		VACV-GFP-24h: EV vs TBIFN-β		<0.001
		VACV-GFP-36h: EV vs TBIFN-β		<0.001
Figure 5	A	PKR: EV vs TBIFN-β	two-way ANOVA followed by Sidak's multiple-comparisons test	0.02
		OAS1: EV vs TBIFN-β		<0.001
		Mx-1: EV vs TBIFN-β		0.11

	B	PKR: EV vs TBIFN-β	two-way ANOVA followed by Sidak's multiple-comparisons test	0.006
		OAS1: EV vs TBIFN-β		<0.001
		Mx-1: EV vs TBIFN-β		>0.99
	C	PKR: EV vs TBIFN-β	two-way ANOVA followed by Sidak's multiple-comparisons test	0.84
		OAS1: EV vs TBIFN-β		<0.001
		Mx-1: EV vs TBIFN-β		0.03
	E	PKR: EV vs TBIFN-β	two-way ANOVA followed by Sidak's multiple-comparisons test	0.74
		OAS1: EV vs TBIFN-β		<0.001
		Mx-1: EV vs TBIFN-β		0.04
Figure 6	A	EV: DMSO vs Pyridone 6	two-way ANOVA followed by Sidak's multiple-comparisons test	0.88
		TBIFN-β: DMSO vs Pyridone 6		<0.001
		DMSO: EV vs TBIFN-β		<0.001
		Pyridone 6: EV vs TBIFN-β		0.01
	B	EV: DMSO vs Pyridone 6	two-way ANOVA followed by Sidak's multiple-comparisons test	>0.99
		TBIFN-β: DMSO vs Pyridone 6		<0.001
		DMSO: EV vs TBIFN-β		<0.001
		Pyridone 6: EV vs TBIFN-β		<0.001
	C	EV: DMSO vs Pyridone 6	two-way ANOVA followed by Sidak's multiple-comparisons test	0.98
		TBIFN-β: DMSO vs Pyridone 6		<0.001
		DMSO: EV vs TBIFN-β		<0.001
		Pyridone 6: EV vs TBIFN-β		0.20
	D	EV: DMSO vs Pyridone 6	two-way ANOVA followed by Sidak's multiple-comparisons test	0.67
		TBIFN-β: DMSO vs Pyridone 6		<0.001
		DMSO: EV vs TBIFN-β		0.005
		Pyridone 6: EV vs TBIFN-β		0.60
	E	EV: DMSO vs Pyridone 6	two-way ANOVA followed by Sidak's multiple-comparisons test	0.27
		TBIFN-β: DMSO vs Pyridone 6		0.002
		DMSO: EV vs TBIFN-β		<0.001
		Pyridone 6: EV vs TBIFN-β		<0.001
	F	EV: DMSO vs Pyridone 6	two-way ANOVA followed by Sidak's	<0.001
		TBIFN-β: DMSO vs Pyridone 6		<0.001
		DMSO: EV vs TBIFN-β		<0.001

		Pyridone 6: EV vs TBIFN-β	multiple-comparisons test	<0.001
	G	EV: DMSO vs Pyridone 6	two-way ANOVA followed by Sidak's multiple-comparisons test	0.001
		TBIFN-β: DMSO vs Pyridone 6		<0.001
		DMSO: EV vs TBIFN-β		<0.001
		Pyridone 6: EV vs TBIFN-β		0.003
	H	EV: DMSO vs Pyridone 6	two-way ANOVA followed by Sidak's multiple-comparisons test	0.44
		TBIFN-β: DMSO vs Pyridone 6		0.001
		DMSO: EV vs TBIFN-β		<0.001
		Pyridone 6: EV vs TBIFN-β		0.79
	I	EV: DMSO vs Pyridone 6	two-way ANOVA followed by Sidak's multiple-comparisons test	0.61
		TBIFN-β: DMSO vs Pyridone 6		<0.001
		DMSO: EV vs TBIFN-β		<0.001
		Pyridone 6: EV vs TBIFN-β		0.88
	J	EV: DMSO vs Pyridone 6	two-way ANOVA followed by Sidak's multiple-comparisons test	0.69
		TBIFN-β: DMSO vs Pyridone 6		0.004
		DMSO: EV vs TBIFN-β		0.001
		Pyridone 6: EV vs TBIFN-β		0.30
	K	EV: DMSO vs Pyridone 6	two-way ANOVA followed by Sidak's multiple-comparisons test	0.79
		TBIFN-β: DMSO vs Pyridone 6		0.04
		DMSO: EV vs TBIFN-β		<0.001
		Pyridone 6: EV vs TBIFN-β		<0.001
Figure 8	D	EV vs TBIFN-β	two-tailed unpaired Student's t-test	0.006
	E	EV vs TBIFN-β		0.03
	F	EV vs TBIFN-β		0.008
	G	EV vs TBIFN-β		0.003
	H	EV vs TBIFN-β		0.003
	I	EV vs TBIFN-β		0.005
Supplementary Figure S4	A	6hpt: 0 vs 0.5μg/mL	one-way ANOVA followed by Dunnett's multiple-comparisons test	0.04
		6hpt: 0 vs 1μg/mL		<0.001
		12hpt: 0 vs 0.5μg/mL		0.50

		12hpt: 0 vs 1μg/mL	one-way ANOVA followed by Dunnett's multiple-comparisons test	<0.001
		24hpt: 0 vs 0.5μg/mL	one-way ANOVA followed by Dunnett's multiple-comparisons test	0.43
		24hpt: 0 vs 1μg/mL		<0.001
	B	6hpt: 0 vs 0.5μg/mL	one-way ANOVA followed by Dunnett's multiple-comparisons test	<0.001
		6hpt: 0 vs 1μg/mL		<0.001
		12hpt: 0 vs 0.5μg/mL	one-way ANOVA followed by Dunnett's multiple-comparisons test	<0.001
		12hpt: 0 vs 1μg/mL		<0.001
		24hpt: 0 vs 0.5μg/mL	one-way ANOVA followed by Dunnett's multiple-comparisons test	<0.001
		24hpt: 0 vs 1μg/mL		<0.001
	C	6hpt: 0 vs 0.5μg/mL	one-way ANOVA followed by Dunnett's multiple-comparisons test	0.0070
		6hpt: 0 vs 1μg/mL		0.0146
		12hpt: 0 vs 0.5μg/mL	one-way ANOVA followed by Dunnett's multiple-comparisons test	0.0041
		12hpt: 0 vs 1μg/mL		0.0088
		24hpt: 0 vs 0.5μg/mL	one-way ANOVA followed by Dunnett's multiple-comparisons test	0.0576
		24hpt: 0 vs 1μg/mL		0.0011
Supplementary Figure S6	A	PKR: EV vs TBIFN-β	two-way ANOVA followed by Sidak's multiple-comparisons test	0.65
		OAS1: EV vs TBIFN-β		0.89
		Mx-1: EV vs TBIFN-β		0.86
	B	PKR: EV vs TBIFN-β		>0.99
		OAS1: EV vs TBIFN-β		0.04
		Mx-1: EV vs TBIFN-β		>0.99
Supplementary Figure S7	C	EV: DMSO vs Pyridone 6	two-way ANOVA followed by Sidak's	0.96
		TBIFN-β: DMSO vs Pyridone 6		<0.001

		DMSO: EV vs TBIFN-β	multiple-comparisons test	<0.001
		Pyridone 6: EV vs TBIFN-β		<0.001
	D	EV: DMSO vs Pyridone 6	two-way ANOVA followed by Sidak's multiple-comparisons test	0.14
		TBIFN-β: DMSO vs Pyridone 6		<0.001
		DMSO: EV vs TBIFN-β		<0.001
		Pyridone 6: EV vs TBIFN-β		0.008
	E	EV: DMSO vs Pyridone 6	two-way ANOVA followed by Sidak's multiple-comparisons test	>0.99
		TBIFN-β: DMSO vs Pyridone 6		<0.001
		DMSO: EV vs TBIFN-β		<0.001
		Pyridone 6: EV vs TBIFN-β		>0.99

For ANOVA-based multiple comparisons, multiplicity-adjusted P values are reported. For two-tailed unpaired Student's t-tests, unadjusted P values are reported.

Supplementary Table S4. Stability of β-actin Ct values under VSV-GFP and VACV-GFP infection.

Condition	Time	β-actin mean Ct	SD
VSV-GFP	0 h	16.977	0.057
VSV-GFP	6 h	16.975	0.009
VSV-GFP	12 h	16.590	0.085
VSV-GFP	24 h	16.946	0.051
VSV-GFP	36 h	17.055	0.114
VACV-GFP	0 h	15.374	0.138
VACV-GFP	6 h	15.238	0.067
VACV-GFP	12 h	15.536	0.084
VACV-GFP	24 h	15.998	0.004
VACV-GFP	36 h	15.228	0.078

Values are presented as the mean ± SD from three independent biological experiments (n = 3).

Supplementary Table S5. Validation of RT-qPCR primer amplification efficiencies.

Gene	Slope	R^2	Efficiency (%)
β-actin	-3.443	0.9963	95.2
TBIFN-β	-3.436	0.9935	95.5
PKR	-3.163	0.9925	107.1
OAS1	-3.405	0.9990	96.7
Mx-1	-3.309	0.9990	100.5
STAT1	-3.381	0.9997	97.6
IRF7	-3.126	0.9977	108.9
MDA5	-3.540	0.9934	91.6
ISG15	-3.353	0.9993	98.7
IFN-ω	-3.419	0.9969	96.1
RANK	-3.584	0.9957	90.1
